# Marburg Virus Glycoprotein Is a Remarkable Virulent Factor Linked to Hemorrhagic Pathology: Evidence from Multimodal Experimental Systems

**DOI:** 10.1002/advs.202511575

**Published:** 2026-04-24

**Authors:** Ting Yao, Hang Liu, Yanfeng Yao, Wei Deng, Jiawen Sun, Zhenyu Kang, Ashaq Ali, Chao Shan, Zhiming Yuan, Fei Deng, Hualin Wang, Yun‐Jia Ning

**Affiliations:** ^1^ State Key Laboratory of Virology and Biosafety Wuhan Institute of Virology Chinese Academy of Sciences Wuhan China; ^2^ National Virus Resource Center Wuhan Institute of Virology Chinese Academy of Sciences Wuhan China; ^3^ University of Chinese Academy of Sciences Beijing China; ^4^ State Key Laboratory of Virology and Center for Biosafety Mega‐Science Chinese Academy of Sciences Wuhan China; ^5^ Center of Excellence in Science and Applied Technologies Islamabad Pakistan; ^6^ Hubei Jiangxia Laboratory Wuhan China

**Keywords:** Ebola virus (EBOV), filovirus, glycoprotein (GP), Marburg virus (MARV), mucin‐like domain (MLD), research models, vascular permeability, viral hemorrhagic fever

## Abstract

Marburg virus (MARV) causes Marburg virus disease (MVD), a severe hemorrhagic fever associated with high morbidity and mortality. No specific antiviral therapies are currently approved for MVD, and viral pathogenesis, particularly the viral pathogenic factor(s) involved, remains poorly defined. Here, we demonstrated that MARV glycoprotein (GP) can induce rounding and detachment of adherent cells, likely by shielding and downregulating cell surface molecules, mainly via its mucin‐like domain (MLD), disrupting monolayer barrier function. In an ex vivo rat vascular model, GP expression caused significant endothelial cell damage and increased vascular permeability. In vivo, MARV GP increases vascular permeability and exacerbates inflammation and tissue injury in mouse muscle and liver transduction models, validating its pathogenic activity. Deletion of the MLD largely abolished these pathogenic effects, indicating its significant role in MARV GP pathogenicity. In addition, using the ex vivo vascular model, we demonstrated the infectivity and pathogenicity of the authentic Ebola virus in vessels and validated the utility of this model as a tool for intervention research against filovirus infection and GP pathogenicity. This study uncovered the remarkable role of MARV GP as a hemorrhage‐related pathogenic factor through multimodal analyses, which may help advance our understanding of viral pathogenesis and lay a foundation for future therapeutic strategies.

## Graphical abstract

By integrating data from in vitro, ex vivo, and in vivo models, our research identifies the MARV glycoprotein as a remarkable hemorrhagic factor, filling a major gap in this important field. It also provides practical experimental tools for the basic research on viral pathogenesis and applied research aimed at antiviral intervention for hemorrhagic fevers.

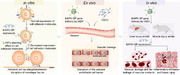

## 1 Introduction

The Marburg virus (MARV) is a highly virulent Biosafety Level‐4 (BSL‐4) pathogen that causes Marburg virus disease (MVD), a severe hemorrhagic fever with a fatality rate of up to 88% [1, 2, 3, 4, 5, 6]. MARV was first identified in 1967 in Marburg, Germany, from which the virus derives its name [7]. MARV is primarily transmitted to humans through direct contact (through broken skin or mucous membranes) with the bodily fluids of infected individuals or through exposure to infected fruit bats, which are considered natural reservoirs [8]. There are no approved antiviral treatments specifically for MARV, and experimental therapies and vaccines are currently under investigation [9, 10]. In recent years, increased global communication has contributed to frequent epidemic outbreaks in various countries and regions, posing great challenges for early warning, prevention, and control. Multiple countries (including Equatorial Guinea, Tanzania, and Rwanda) have recently reported the first‐ever outbreak of MVD [11, 12, 13, 14, 15], causing significant loss of life and property, as well as widespread panic [12, 16, 17, 18, 19]. Furthermore, the high fatality rate and lack of medical resources exacerbate the threat to public health. Accordingly, the World Health Organization (WHO) has listed MARV as a “priority pathogen” demanding extensive attention and urgent research [20].

MVD begins abruptly, presenting with high fever, severe headache, malaise, and muscle aches, and is characterized by significant bleeding, which is closely linked to viral fatalities [5, 18, 21, 22]. Vascular injury, especially in endothelial cells, is a significant contributor to hemorrhage [23]. Endothelial cells play a crucial role in maintaining vascular barrier function; when damaged, this barrier is compromised, leading to direct vascular leakage [24]. In addition, vascular endothelial cells regulate platelet function, further influencing coagulation [24, 25]. Furthermore, MARV infection induces the upregulation of various inflammatory cytokines that can adversely affect blood vessels, particularly endothelial cells, intensifying vascular dysfunction [26]. Presumably, direct viral injury to blood vessels, inflammatory responses, and coagulation dysfunction interact synergistically, mutually exacerbating the overall hemorrhagic process. Moreover, MARV can infect various organs and tissues, leading to multiple organ failure, with liver damage being a significant clinical hallmark [18]. The liver synthesizes most coagulation factors in the human body and plays an essential regulatory role in maintaining the dynamic balance between coagulation and anticoagulation. The severity of liver dysfunction often parallels that of coagulation disorders [27]. In addition, the liver possesses robust immune functions that can eliminate invading pathogens through phagocytosis [27, 28]. Consequently, liver damage profoundly affects both the stability of the circulatory system and the normal functioning of the immune system. The pathogenesis of MARV infection is highly complex, and the aforementioned effects represent hypothetical inferences regarding the mechanisms underlying viral hemorrhagic fever. Specific viral pathogenic factors, especially those involved in vascular injury, liver dysfunction, and resultant hemorrhage, remain unknown. Beyond MVD, the pathological factors and mechanisms by which hemorrhagic fever viruses (HFVs) induce hemorrhage are shrouded in mystery. The stringent requirements for high‐containment biosafety facilities to handle these highly pathogenic viruses, together with the scarcity of available experimental models, further impede the study of their pathogenesis and the development of antiviral therapeutics.

Similar to the Ebola virus (EBOV), MARV is a negative‐sense, single‐stranded, non‐segmented RNA virus belonging to the *Filoviridae* family [6, 29]. The MARV genome is approximately 19 kb in length and encodes multiple proteins. Glycoprotein (GP) is synthesized as a 681‐amino acid type I transmembrane protein and is cleaved into two disulfide‐linked subunits, GP_1_ and GP_2_, by the host cell pro‐protein convertase furin after translation [30, 31]. A notable feature of both MARV and EBOV GPs is their extensive O‐ and N‐glycosylation [32]. In the middle of the GPs, there is a high concentration of the glycosylation region, known as the mucin‐like domain (MLD). Despite being members of the *Filoviridae* family, MARV and EBOV belong to different viral genera. Their GPs share only 30% amino acid sequence identity, and there is no apparent sequence identity with MLDs [33, 34, 35, 36]. Moreover, the GP expression strategies of MARV and EBOV differ substantially [37]. EBOV primarily expresses a soluble GP (sGP) lacking a transmembrane domain, while the full‐length transmembrane GP—capable of anchoring to the plasma membrane—is encoded via transcriptional editing (resulting in a +1 frameshift) and accounts for only about 24% of total GP transcripts. Besides, a +2 frameshift during transcriptional editing (approximately 5% of transcripts) generates a small sGP (designated ssGP). Furthermore, the surface GP of EBOV can be proteolytically cleaved to release a shed GP. In contrast, MARV GP is expressed directly without transcriptional editing and does not produce these soluble GP isoforms. In addition, the different positions of the furin cleavage site result in the entire MLD being attached to GP_1_ in EBOV, whereas it is cleaved into two parts attached to GP_1_ and GP_2_ in MARV [36, 38]. Previous studies have demonstrated that EBOV GP can form a spatial shield through its MLD, masking host molecules on the cell surface and inducing morphological changes in adherent cells in vitro [39, 40, 41, 42, 43]. Through adenovirus (ADV) vector transduction, EBOV GP expression was shown to increase vascular permeability in ex vivo models [48], and induce distinct cell and tissue damage, as well as acute inflammation in a mouse muscle injury model established in our laboratory [39]. The shed GP of EBOV has also been suggested to potentially induce immune evasion, stimulate inflammation, and increase cell monolayer permeability [44, 45]. These effects are likely linked to viral pathogenesis, although the infectivity and pathogenicity of EBOV in vascular tissues remain poorly understood. However, it is unclear whether MARV GP (or MLD) exhibits similar functionality, particularly given the low sequence homology, divergent post‐translational processing, and absence of soluble GP isoforms.

In this study, we systematically investigated and demonstrated the pathogenicity of MARV GP using in vitro, ex vivo, and in vivo models for the first time. Meanwhile, the MLD of MARV GP is required for pathogenic effects. Moreover, by leveraging the newly developed ex vivo rat vascular hemorrhagic model, we confirmed the vascular infectivity and pathogenicity of authentic EBOV and validated the model's utility as a platform for studies on targeted pharmacological interventions against filovirus infection and GP pathogenicity.

## 2 Results

### 2.1 MARV GP Induces Cell Rounding and Detachment and Disrupts the Barrier Function of Adherent Cell Monolayers, Which Requires Its MLD

**FIGURE 1:**
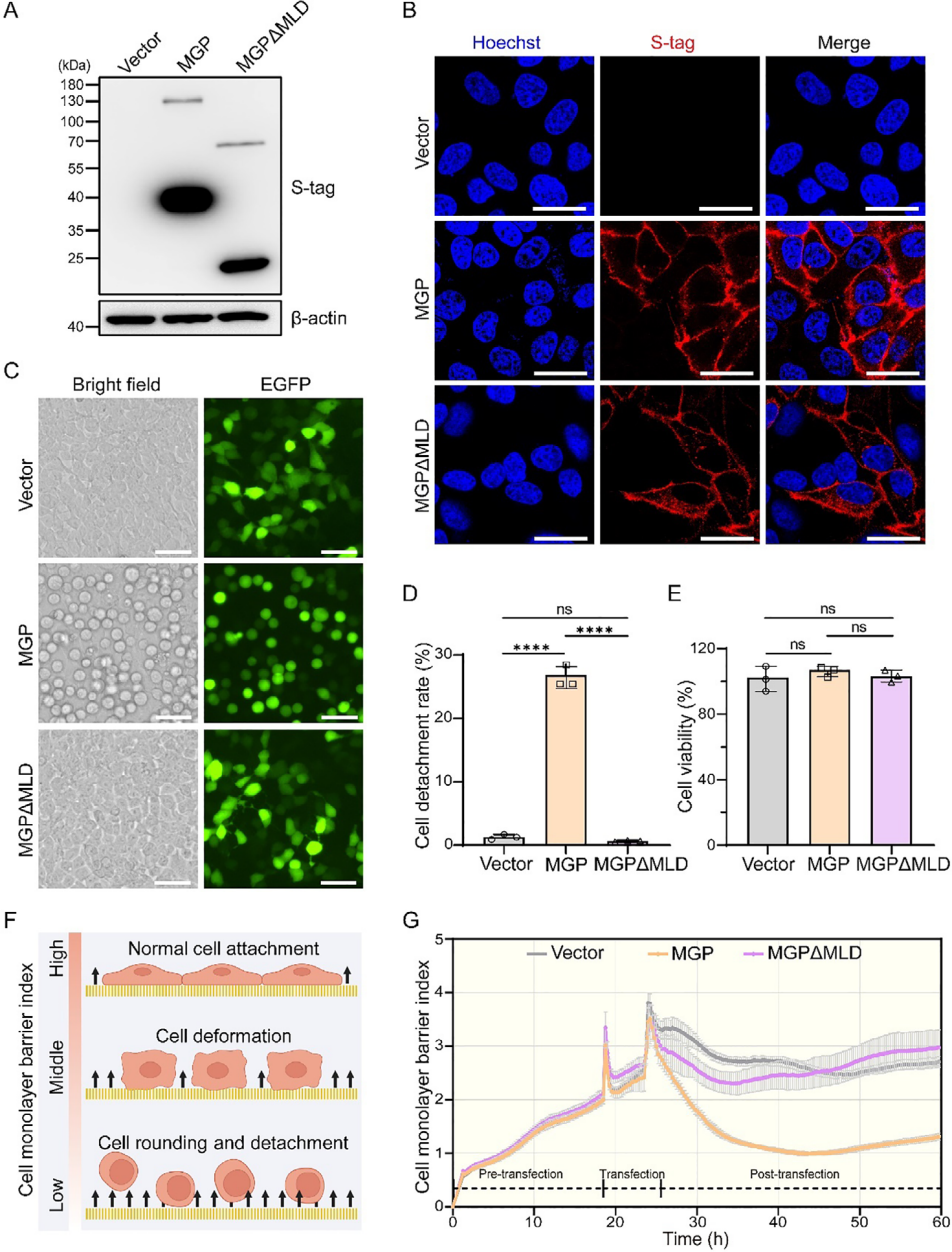
MARV GP (MGP), but not the mucin‐deleting mutant (MGPΔMLD), induces cell rounding/detachment and disrupts the barrier function of the cell monolayer. (A) HEK293T cells were transfected with plasmids expressing full‐length (MGP) or MLD‐deleted (MGPΔMLD) MARV GPs. At 24 h post‐transfection, protein expression was analyzed by western blotting (WB) using the indicated antibodies. Original images are shown in Figure S13A,B. (B) HeLa cells transfected with plasmids encoding the indicated proteins or the control vector were fixed and permeabilized, followed by immunofluorescence assays (IFA) with anti‐S‐tag antibody and confocal microscopy 24 h post‐transfection. Nuclei stained with Hoechst are represented in blue. Scale bars, 25 µm. (C) HEK293T cells were co‐transfected with the indicated protein expression plasmids or the control vector along with pEGFP‐N1, followed by visualization 24 h later under an inverted fluorescence microscope. Merged images are shown in Figure S14A. Scale bars, 50 µm. (D) Quantitative analysis of the cell detachment. HEK293T cells were transfected with MGP, MGPΔMLD expression plasmids, or control vectors. At 24 h post‐transfection, the detached and total cells were counted, and the detachment rates were calculated. Data are presented as mean ± SD, *n* = 3 biological replicates. One‐way analysis of variance (ANOVA) was used for multiple comparisons. ^****^ *p <* 0.0001; ns, nonsignificant. (E) HEK293T cells were transfected with the indicated expression plasmids and controls. At 24 h post‐transfection, total cells (including adherent and detached cells) were collected to analyze cell viability using the CCK‐8 assay. Data are presented as mean ± SD, *n* = 3 biological replicates. One‐way analysis of variance (ANOVA) was used for multiple comparisons. ns, nonsignificant. (F) Schematic representation of cell monolayer permeability detection using RTCA. In the RTCA‐based analysis, the barrier capability of the cell monolayer was positively correlated with the impedance between the two electrodes. Thus, disruption of the cell monolayer barrier by cell deformation, rounding, or even detachment leads to a decrease in impedance and an increase in ion flow (black arrows) to different degrees. This correlation allows the real‐time detection of the barrier function or permeability of the cell monolayer using an RTCA system. Created in BioRender. Yao, T. (2026) https://BioRender.com/moqtvri. (G) HEK293T cells were seeded in an RCTA E‐plate and transfected with the indicated expression plasmids or control vector. The cell barrier function was assessed in real time using RCTA. Each curve represents the mean and SD of three independent biological replicates.

To investigate the effects of MARV GP and its MLD on adherent cells, two expression plasmids were constructed to produce wild‐type MARV GP (MGP) and a variant lacking the MLD (MGPΔMLD), both with an S‐tag fused to the C‐terminus. The expression and cell membrane localization of viral proteins were confirmed using western blotting (WB) and immunofluorescence assays (IFA) (Figure 1A,B). During these analyses, MGP slightly deformed HeLa cells (mainly manifested as a tendency to contract and become round). Thus, we further investigated the potential influence of MGP on the adherence of HEK293T cells, which are relatively sensitive to factors affecting cellular attachment and adhesion [46]. MGP expression in adherent HEK293T cells induced cell rounding and detachment (Figure 1C,D). The effects of MGP on cell morphology and adherence were abolished by deletion of the MLD (Figure 1C,D). When the detached cells were collected for continued culture, they remained viable and gradually regained adhesion, however, the time required for re‐attachment was much longer than that of control cells (Figure S1A). Consistently, the viability of the cells expressing MGP or MGPΔMLD was not significantly affected, as validated by CCK‐8 assays and trypan blue staining analysis (Figure 1E; Figure S1B). Similarly, the viral protein expression did not impair the survival of other cell types either, including HeLa, HUVEC, and Huh7 cells (Figure S1C–E). These findings suggest that the cytopathogenicity caused by MGP manifests as cell rounding and loss of adhesion rather than toxicity, leading to cell death. Thus, cells likely regained adhesive properties gradually as the transient expression diminished. Furthermore, we considered that cell deformation and adhesion impairment induced by MGP may compromise the barrier function of adherent cell monolayers and enhance permeability, which might be intricately associated with the viral pathogenesis mechanism. To validate this hypothesis, we first monitored the impact of MGP on the barrier function/permeability of E‐plate monolayer adherent cells using a Real‐Time Cell Analyzer (RTCA) [47] (Figure 1F). MGP substantially increased the permeability of the adherent cell layer, severely disrupting its barrier function (Figure 1G). In contrast, the expression of the protein with MLD deletion failed to exert such an effect, demonstrating a cell monolayer barrier activity comparable to that of the negative control group (Figure 1G). These results establish that MARV GP can induce rounding and detachment of cultured adherent cells, disrupting the barrier function of the adherent cell layer. Moreover, MLD is required for these effects.

### 2.2 MGP Interferes with Cell Surface Molecules, Likely Not Only by Glycan Shielding But also by Downregulating Their Total Expression Levels, Both of Which Largely Depend on the MLD

**FIGURE 2:**
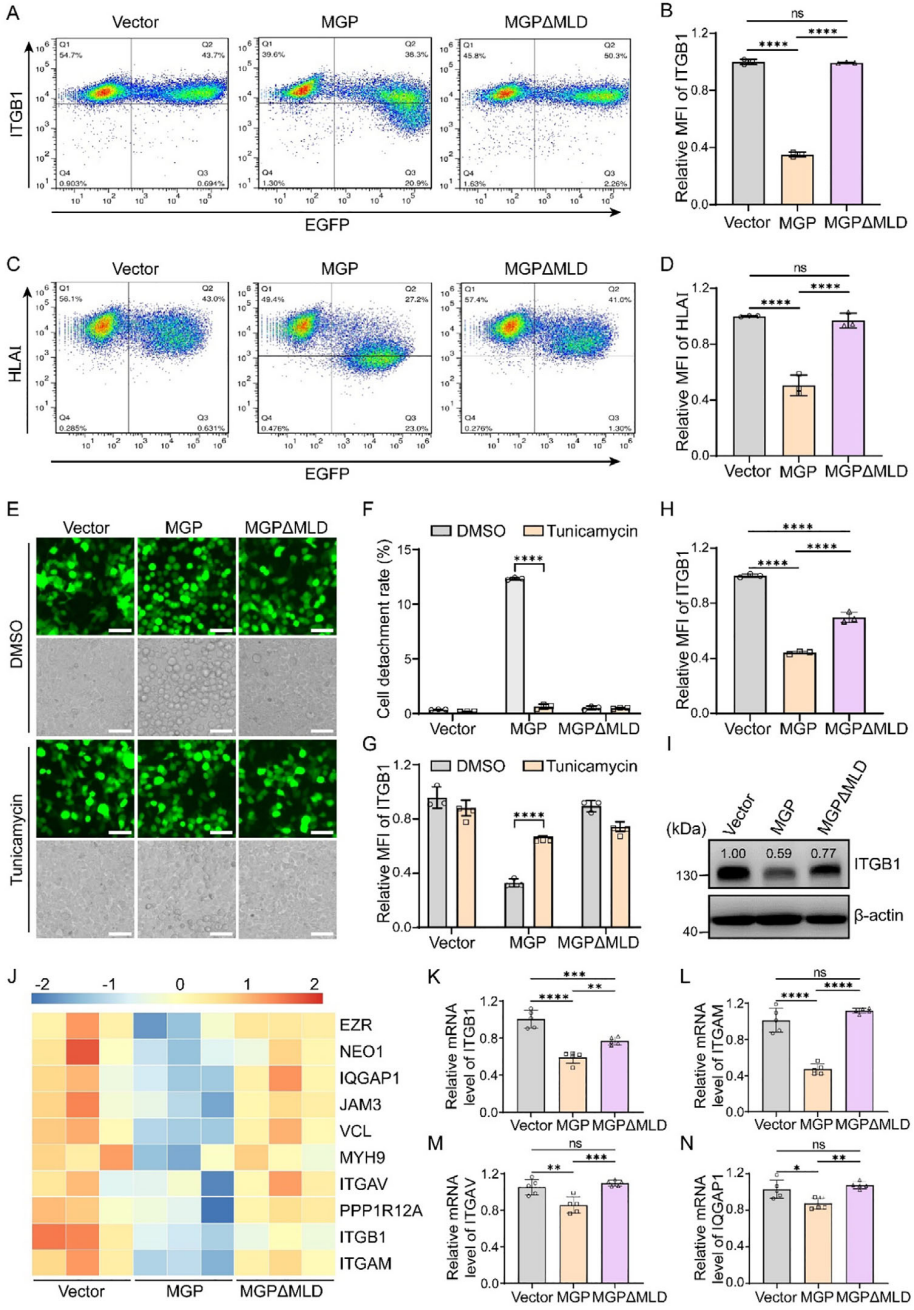
MGP downregulates the detectable signals on the cell membrane by IFA and FCM and the total expression levels of cell surface molecules. (A–D) MGP (but not MGPΔMLD) reduced the detectable signals of ITGB1 and HLA‐I on the cell surface. HEK293T cells were co‐transfected with the indicated protein expression plasmids or control vectors along with pEGFP‐N1, followed by IFA of non‐permeabilized cells with PE‐conjugated anti‐ITGB1 (A, B) or anti‐HLA‐I (C, D) antibodies and flow cytometry (FCM) analysis. Scatter plots (A, C) and mean fluorescence intensities (MFI) (B, D) of the cell surface protein signals are shown. Data are presented as mean ± SD, *n* = 3 biological replicates. One‐way analysis of variance (ANOVA) was used for multiple comparisons. ^****^ *p <* 0.0001; ns, nonsignificant. (E) HEK293T cells co‐transfected with the indicated protein expression plasmids or control vector, along with pEGFP‐N1 were treated with tunicamycin (2 µg/mL) or the solvent DMSO control immediately after transfection. After 24 h, the cells were visualized using an inverted fluorescence microscope. Merged images are shown in Figure S14B. Scale bars, 50 µm. (F) HEK293T cells transfected with the indicated expression plasmids or control vector were treated with tunicamycin (2 µg/mL) or DMSO, followed by quantification of cell detachment 24 h post‐transfection. Data are presented as mean ± SD, *n* = 3 biological replicates. Student's t‐test was used for comparisons between the two groups. ^****^ *p <* 0.0001. (G) HEK293T cells were co‐transfected with the indicated expression plasmids or control along with pEGFP‐N1 and treated with tunicamycin (2 µg/mL) or DMSO for 24 h, followed by IFA and FCM analyses with non‐permeabilized cells. Data are presented as mean ± SD, *n* = 3 biological replicates. Student's t‐test was used for comparisons between the two groups. ^****^ *p <* 0.0001. (H) HEK293T cells were co‐transfected with the indicated expression plasmids or control vectors along with pEGFP‐N1. After 24 h, the cells were collected for fixation and permeabilization, followed by IFA. The MFI of total ITGB1 in EGFP‐positive cells was analyzed using FCM. Data are presented as mean ± SD, *n* = 3 biological replicates. One‐way analysis of variance (ANOVA) was used for multiple comparisons. ^****^ *p <* 0.0001. (I) HEK293T cells transfected with the indicated plasmids were subjected to WB analysis 24 h after transfection. Original images are shown in Figure S13C. The relative band intensities of ITGB1 were quantified using ImageJ software. (J) Heatmap displaying log_2_FC for 10 key genes related to the intracellular adhesion of transfected HEK293T cells. Three biological replicates were performed under each condition. (K–N) qPCR analysis of mRNA levels of representative cell‐adhesion proteins. Data are presented as mean ± SD, *n* = 5 biological replicates. One‐way analysis of variance (ANOVA) was used for multiple comparisons. ^****^ *p <* 0.0001; ^***^ *p <* 0.001; ^**^ *p <* 0.01; ^*^ *p <* 0.05; ns = not significant.

Given the deadhesion activity of MGP and its high glycosylation, we hypothesized that MGP might also mask cell surface adhesion molecules via its heavy glycans, especially in MLD, causing cell rounding and adhesion impairment. Therefore, we examined the effect of MGP expression on cell surface molecules using IFA in non‐permeabilized HEK293T cells. Consistent with our hypothesis, MGP significantly reduced the detectable signals of the representative adhesion molecule integrin‐β1 (ITGB1) and immune response molecule HLA‐I on the cell surface (Figure 2A–D). However, this effect was barely observable after deletion of the MLD (Figure 2A–D). Consistently, treatment with a glycosylation inhibitor, tunicamycin, suppressed MGP‐induced cell rounding and detachment and significantly reversed the shielding effect of MGP on the cell surface molecule ITGB1 (Figure 2E–G). These results suggest that MGP may sterically mask cell surface molecules via its MLD glycan cap [33, 34, 35, 36], leading to changes in cell morphology and loss of adhesion.

Consistent with a cell surface‐shielding mechanism, deletion of either the signal peptide (disrupting ER‐Golgi trafficking, glycosylation, and membrane localization) or the transmembrane domain (impairing plasma membrane anchoring) abolished MGP‐induced cell rounding, detachment, and ITGB1 masking (Figure S2), underscoring the necessity of surface expression. Although the MLD is essential, MLD constructs—even those artificially targeted to the plasma membrane—failed to recapitulate MGP's effects (Figure S3), indicating an additional requirement for the full‐length protein context. Furthermore, point mutations of individual glycosylation sites did not significantly impair function, suggesting that the dense glycosylation pattern as a whole, rather than some specific sites, may mediate the spatial shielding. Deletion of the MLD N‐terminal region impaired the MGP's activity more severely than C‐terminal deletion, highlighting its particularly important role (Figure S4). Additionally, infection with pseudoviruses bearing full‐length MGP—even though they contain the complete MGP form—did not induce any cell rounding or detachment (Figure S5), further supporting the mechanism that MGP needs to be expressed on the plasma membrane to mediate steric shielding of cell‑surface adhesion molecules. Therefore, optimal disruption of cell adhesion by MGP likely depends on its cell surface expression, MLD, broad glycosylation, and full‐length protein context.

IFA and WB analyses of total ITGB1 protein levels in permeabilized cells and whole‐cell lysates showed that MGP downregulated the total expression of this adhesion molecule in HEK293T cells (Figure 2H,I). This is in contrast to the effect of EBOV GP reported previously (EBOV GP does not seem to affect the expression of cell surface molecules) [43]. To further investigate the unique effect of MGP, we conducted transcriptome sequencing analysis of cells expressing MGP and MGPΔMLD. The results showed that MGP downregulated the expression of numerous cell adhesion‐related molecules, and deletion of the MLD reversed this effect on most adhesion‐related molecules to varying degrees (Figure 2J; Figure S6). Quantitative PCR (qPCR) further validated these findings (Figure 2K–N). Furthermore, transcriptomic analyses reveal that numerous genes involved in endoplasmic reticulum (ER) stress and the unfolded protein response (UPR) are differentially regulated by MGP, underscoring the multifaceted and complex roles of this viral glycoprotein in modulating host cellular homeostasis (Figure S7).

Additionally, similar effects—where MGP (but not MGPΔMLD) reduces surface detectable signal and total protein expression of adhesive molecule ITGB1—were also observed in other human cells, including HUVECs (endothelial cells) and Huh7 (epithelial cells) (Figure S8A–D). Consistently, qPCR results further supported a downward trend in the overall transcriptional expression of various adhesion factors (as well as HLAs) in vascular endothelial cells (HUVECs) induced by MGP (Figure S8E–L). These observations suggest that MGP may exacerbate adhesion loss by nonspecifically downregulating the expression of numerous adhesion‐related molecules. Moreover, MLD likely contributes to the effect of MGP, although it does not appear to be the sole cause.

### 2.3 MGP Induces Vascular Endothelial Cell Injury and Increases Vascular Permeability via Its MLD in an ex vivo Vessel Model

**FIGURE 3:**
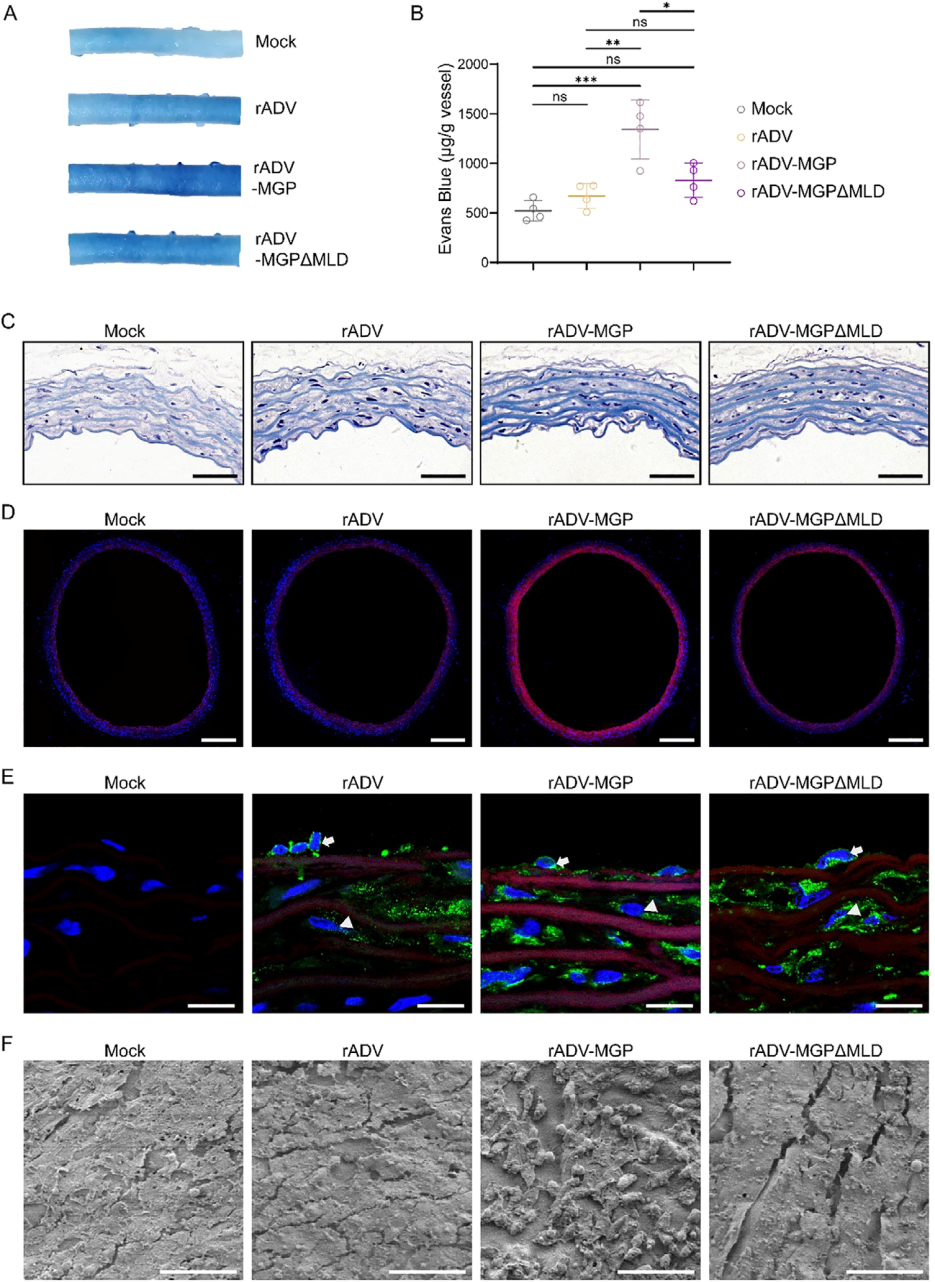
MGP induces vascular endothelial cell injury and increases vascular permeability in an ex vivo blood vessel model, which can be largely attributed to MLD. (A) Rat aortic vessels were cultured ex vivo and transduced with the indicated adenoviral vectors. After 48 h, vessel permeability was evaluated using Evans blue (EB) staining. (B) The darker the color of the EB dye, the poorer the barrier function (i.e., higher permeability) of the vessels. EB from the stained vessels was extracted with formamide and quantitatively analyzed using the standard curve method. Data are presented as mean ± SD, *n* = 4 biological replicates. One‐way analysis of variance (ANOVA) was used for multiple comparisons. ^***^ *p <* 0.001; ^**^ *p <* 0.01; ^*^ *p <* 0.05; ns = not significant. (C) Paraffin‐embedded sections of EB‐stained vessels were prepared and observed under a light microscope after hematoxylin staining. (D) In parallel experiments, the EB signals (with a strong absorption peak at 540 nm) of the paraffin‐embedded sections were visualized using fluorescence confocal microscopy and are shown in red. Nuclei (blue) were stained with Hoechst 33258. (E) After IFA, viral transduction in endothelial cells (arrows) and vascular smooth muscle cells (arrowheads) was observed using confocal microscopy. (F) Scanning electron microscopy (SEM) revealed endothelial cell rounding and detachment from the underlying basement membrane, specifically after transduction with the full‐length MGP‐expressing vector (rADV‐MGP), but not after MLD‐deleting or other controls. Scale bars, 50 µm (C and F), 200 µm (D), or 25 µm (E).

As a typical hemorrhagic fever virus, MARV infection can cause severe clinical bleeding symptoms; however, its pathogenic factors and mechanisms remain unclear. Given that MGP can reduce cell adhesion and disrupt the cell monolayer barrier function, we investigated its potential role in MARV pathogenesis using an ex vivo vessel model. To express proteins both ex vivo and in vivo, recombinant adenoviruses (rADV) encoding MGP (rADV‐MGP) or MGPΔMLD (rADV‐MGPΔMLD) were constructed. Consistently, transduction with recombinant viral vectors expressing MGP, but not the MGPΔMLD variant, induced pronounced cell rounding in multiple cell lines, including HEK293 and SW13 cells (Figure S9). To assess the effect of MGP on vasculature, rat aortas were isolated and cultured as an ex vivo transduction model. Vessel permeability was evaluated using Evans Blue (EB) staining. Intriguingly, transduction with rADV‐MGP led to significantly enhanced vascular permeability (as indicated by stronger EB dye intake), whereas adenovirus transduction alone exerted no or minimal effects on vascular permeability (no statistically significant differences were observed in the quantitative analysis) (Figure 3A,B). Moreover, deletion of the MLD (rADV‐MGPΔMLD) significantly abolished MGP‐induced vascular permeability enhancement (Figure 3A,B). Light microscopy analysis of paraffin‐embedded vascular tissue sections further demonstrated increased permeability in the MGP group (Figure 3C). Given that EB exhibits a maximum emission wavelength of 540 nm when excited at 480 nm, we further validated the enhanced vascular permeability caused by MGP using fluorescence microscopy (as evidenced by stronger EB signals in the vascular inner wall, particularly in the elastic fibers) (Figure 3D). IFA and confocal analyses confirmed both MGP‐induced vascular permeability enhancement and the infectivity of the adenoviral vector in vascular tissues (Figure 3E). The viral vectors infected both vascular endothelial cells and vascular smooth muscle cells, which are the main cell types in isolated vessels (Figure 3E). Furthermore, the potential disruptive effects on vessels, particularly vascular endothelial cells, were investigated using scanning electron microscopy (SEM). In the inner walls of vessels, endothelial damage caused by MGP, but not by the MLD‐deletion mutant or other controls, was evident, and the endothelial cells lost their normal morphology, transitioning from a spread and tightly adherent state to a shrunken appearance; the space between cells increased significantly, and some cells even detached from the vessel wall (Figure 3F). These findings suggest that MGP can damage vascular endothelial cells and increase vascular permeability in the presence of MLD, likely contributing to the pathogenic viral process that leads to hemorrhage.

### 2.4 MGP Increases Vascular Permeability, Inflammatory Infiltration, and Tissue Injury via MLD in in vivo Models

To investigate the pathogenicity of MGP in vivo, We first used a previously established mouse muscle (gastrocnemius) transduction model (Figure S10A) [39]. Because the recombinant viral vectors used are replication‐defective and can only facilitate a single infection, the muscle transduction model allows for effective targeting of specific areas, whereas transduction dynamics and clinical pathological changes in the muscle can be conveniently monitored via non‐invasive imaging after hair removal. At 48 h post‐intra‐gastrocnemius injection, the mice inoculated with rADV‐MGP exhibited redness and hyperalgesia to external stimuli in the muscle, whereas the control (Mock, rADV, or rADV‐MGPΔMLD) groups displayed only mild or negligible symptoms. At 72 h post‐injection, EGFP signals localized in the gastrocnemius were detected by live imaging, confirming effective transduction (Figure S10B). Histopathological analysis of the gastrocnemius muscle revealed significant tissue damage in the MGP group, including pyknosis and necrosis of muscle cells, muscle fiber disintegration, and connective tissue hyperplasia in the inflammatory infiltration area (Figure S10C,D). In addition, vascular congestion and hemorrhage (characterized by red blood cell extravasation) in the lesioned region were observed in the rADV‐MGP group (Figure S10C,D). In contrast, tissue damage caused by rADV or rADV‐MGPΔMLD was mild or nearly absent, indicating that pathological injury is associated with MGP expression and the presence of its MLD. Furthermore, both muscle and peripheral connective tissues exhibited substantial inflammatory cell infiltration in the rADV‐MGP group, whereas the rADV and rADV‐MGPΔMLD groups showed only localized mild inflammation (Figure S10E,F).

**FIGURE 4:**
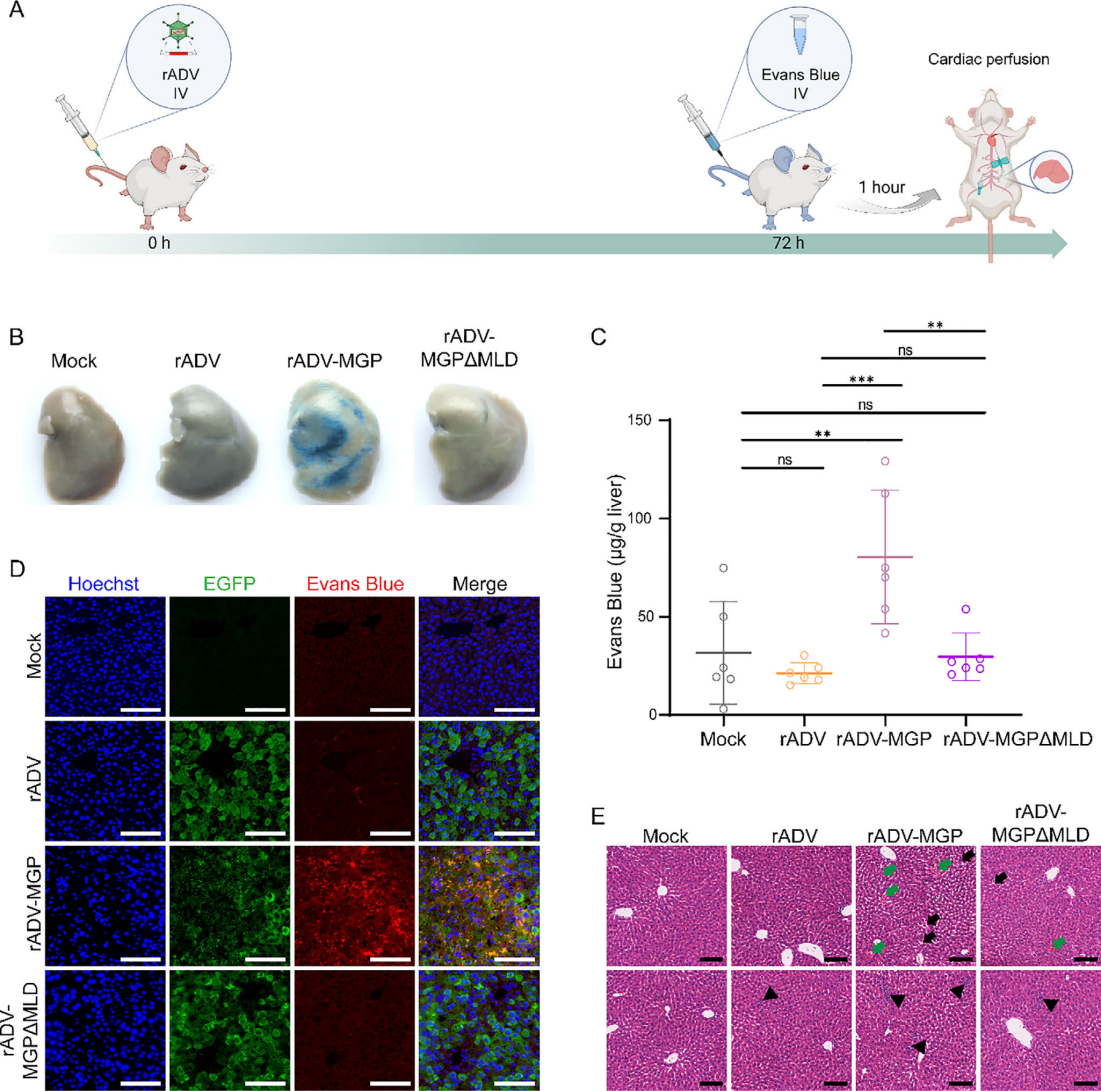
MGP increases vascular permeability, inflammatory infiltration, and tissue injury in the liver in vivo, largely via its mucin‐like domain. (A) Schematic representation of the vascular permeability analysis process in the liver. BALB/c mice were intravenously injected with 100 µL of the indicated recombinant viral vectors (2 × 10^10^ TCID50/mL). After 72 h, EB was administered intravenously and allowed to circulate systemically for 1 h. Subsequently, unabsorbed EB in the blood was removed via cardiac perfusion, followed by anatomical sampling of the liver for further analysis. Created in BioRender. Yao, T. (2026) https://BioRender.com/grird0i. (B–D) MGP, but not the MLD‐deletion mutant, caused leakage of EB into the liver. Distinct blue plaques indicate EB leakage in the liver lobules of the MGP group (but not in the other control groups) (B). EB uptake was quantitatively analyzed after extraction (C) or by confocal microscopy following IFA of paraffin‐embedded liver sections (D). Data are shown as mean ± SD, *n* = 6 mice/group (C). One‐way analysis of variance (ANOVA) was used for multiple comparisons. ^***^ *p <* 0.001; ^**^ *p <* 0.01; ns, nonsignificant. Viral transduction was monitored using an anti‐EGFP reporter antibody; red indicates leakage and uptake of EB in liver tissues. Nuclei stained with Hoechst are shown in blue. (E) H&E pathological analysis of mouse liver sections. Notable pathological changes were also observed. Green arrows indicate bleeding and congestion in the tissue, black arrows indicate tissue damage (mainly manifested as an increase in the space between liver cells), and black arrowheads indicate inflammatory cell infiltration in the liver. Two fields are shown for each group to exhibit the typical pathological changes. Scale bars, 100 µm (D, E).

The liver is the primary target organ of MARV and is critically involved in viral pathogenesis, including hemorrhage development [48]. Thus, we further validated the ability of MGP to increase vascular permeability and investigated its effect on the liver using an in vivo liver transduction model via intravenous injection of MGP‐expressing or control viral vectors (Figure 4A). Intravenous injection is a well‐established method for hepatic delivery, allowing most recombinant adenoviral vectors to be localized in the liver via the bloodstream, although a lower proportion of viruses may reach other organs [49, 50]. At 72 h post‐transduction, EB dye was intravenously injected to enable systemic circulation for one hour. Following euthanasia and complete removal of residual EB from the bloodstream via cardiac perfusion, the livers were excised to evaluate EB extravasation from the vessels into the liver tissues. Compared with the mock‐ and rADV‐treated mice, the rADV‐MGP group exhibited prominent EB plaque deposits in the liver, indicating significant vascular barrier damage (Figure 4B,C). Furthermore, consistent with the data obtained above, MLD removal significantly mitigated the damage to vascular integrity, with EB deposition reduced to levels comparable to those in the control group (Figure 4B,C). Subsequently, panoramic statistical analysis of immunofluorescence signals in paraffin‐embedded liver sections demonstrated effective transduction by all adenoviral vectors, with transduction efficiencies reaching ∼20% (Figure S11). Consistently, an evident staining of EB was specifically observed in the rADV‐MGP group, corroborating the damaging effect of MGP on the blood vessels (Figure 4D). EGFP signals in some local foci appeared to be slightly disrupted in the rADV‐MGP group due to hepatocyte injury and necrosis, as further analyzed by hematoxylin and eosin (H&E) staining. In parallel with the H&E staining of transduced liver sections without EB treatment, significant congestion and erythrocyte exudation, along with hepatocellular swelling and disrupted parenchymal architecture, were specifically observed after rADV‐MGP transduction (Figure 4E). In addition, pronounced Kupffer cell aggregation was observed in the rADV‐MGP group, whereas the rADV and rADV‐MGPΔMLD groups showed no or minimal hemorrhage and Kupffer cell activation (Figure 4E), underscoring the necessity of MLD for MGP‐mediated pathogenic effects in the liver. Together, these data not only validate the capacity of MGP to disrupt the vascular barrier and increase vascular permeability but also suggest the pathogenicity of MGP in liver tissues, including exacerbating tissue injury and inflammation.

Given that MGP promotes immune‐inflammatory cell infiltration in both the aforementioned in vivo liver and muscle models, we further extended our analysis to examine its regulatory effects on the expression of additional immune‐inflammatory mediators, including those potentially associated with endothelial cell activity, in cultured HUVECs. We found that MGP indeed significantly stimulates the upregulation of various inflammatory and immune factors while simultaneously suppressing the basal expression of the anti‐inflammatory cytokine IL‐10 and the antiviral type I IFN in HUVECs (Figure S8M–R). The results align with the pro‐inflammatory activity of MGP observed in the in vivo models. Furthermore, the suppression of antiviral immune factors such as HLAs and IFN‐β would in turn facilitate viral infection. These effects may synergize and exacerbate the pathogenicity of MGP, likely further contributing to viral pathogenesis, including hemorrhagic processes.

### 2.5 EBOV Infection Induces Vascular Endothelial Cell Injury and Increases Vascular Permeability

Previous studies have investigated the vascular‐damaging effects of EBOV GP (EGP) using cultured human or porcine blood vessels combined with recombinant adenoviral‐mediated EGP expression [51]. However, these vascular models face limitations due to restricted tissue availability and high heterogeneity arising from diverse genetic backgrounds. In comparison, the rat vascular model established in this study offers easy accessibility, cost‐effectiveness, and enhanced experimental reproducibility due to the use of genetically homogeneous laboratory animals. Furthermore, although previous studies have solely tested adenovirus‐mediated EGP expression, the pathogenicity of authentic EBOV infection in vessels remains unexplored. Therefore, we used this rat vascular model to investigate the infectivity and pathogenicity of live filoviruses. Given the unavailability of live MARV in our BSL‐4 laboratory, we conducted the following studies using EBOV.

**FIGURE 5:**
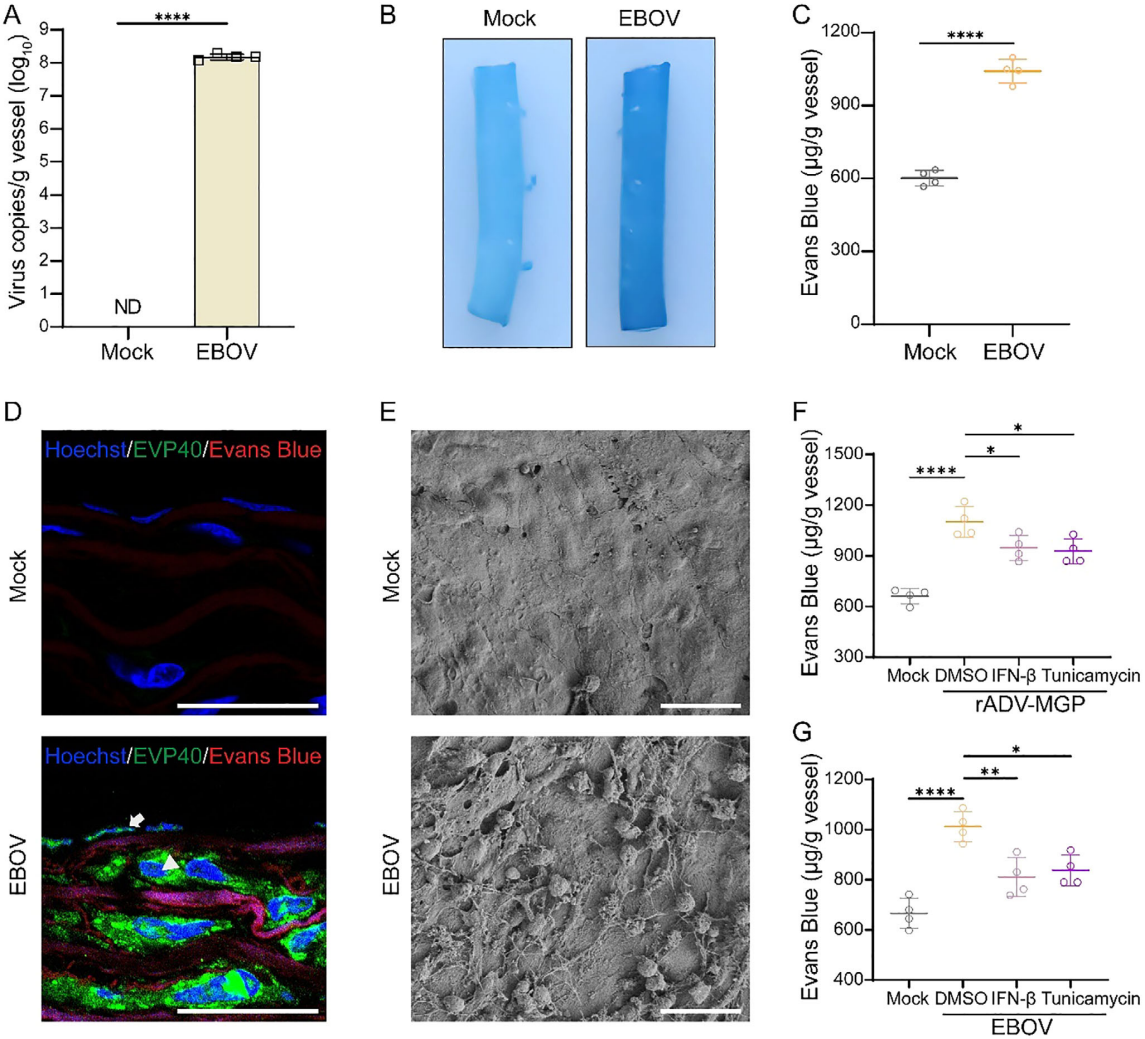
Infection and pathogenicity of authentic live EBOV and evaluation of therapeutic interventions in the ex vivo rat vascular model. (A) Viral load in EBOV‐infected vessel explants. The isolated rat vessels were infected with EBOV. At 48 h post‐infection, the number of viral RNA copies in the infected vessels was determined using qPCR. Data are presented as means ± SD, *n* = 4 biological replicates. Student's *t*‐test was used for comparisons between the two groups. ^****^ *p <* 0.0001; ND, not detected. (B and C) EBOV infection increases vascular permeability. Blood vessels were infected with the EBOV. At 48 h post‐infection, vascular permeability was analyzed using EB staining (B) and quantified after extraction (C). Data are presented as mean ± SD, *n* = 4 biological replicates. Student's *t*‐test was used for comparisons between the two groups. ^****^ *p <* 0.0001. (D) EBOV‐infected and control vessel sections were subjected to IFA with an anti‐EBOV‐VP40 (EVP40) antibody, following EB staining. EBOV infection of vascular cells (green), EB (red), and nuclei (blue) was visualized using confocal microscopy. Arrows indicate infected vascular endothelial cells, and arrowheads indicate infected vascular smooth muscle cells. Scale bars, 25 µm. (E) EBOV infection induces vascular endothelial cell detachment and disrupts the endothelial barrier. Blood vessels were infected with EBOV and subjected to SEM analysis 48 h post‐infection. Scale bars, 25 µm. (F and G). Pharmacological intervention of the increase in vascular permeability induced by rADV‐MGP transduction or EBOV infection. Blood vessels were treated with IFN‐β (500 µg/mL) or tunicamycin (8 µg/mL), along with rADV‐MGP transduction (F) or EBOV infection (G). At 48 h post‐ transduction/infection, the vessels were subjected to EB staining and quantitative analysis. Mock‐infected vessels treated with dimethyl sulfoxide (DMSO) were used as negative controls. Data are presented as mean ± SD, *n* = 4 biological replicates. One‐way analysis of variance (ANOVA) was used for multiple comparisons. ^****^ *p <* 0.0001; ^**^ *p <* 0.01; ^*^ *p <* 0.05.

Prior to live virus experiments, we first validated the pathogenicity of EGP using the rat ex vivo vessel model established herein. The results showed that EGP expression via ADV transduction indeed induced increased vascular permeability (Figure S12). After confirming EBOV infection in vascular tissues via qPCR, EB staining revealed a significant increase in vascular permeability after infection (Figure 5A–C). Subsequently, histological sectioning and IFA further confirmed EBOV infection of vascular endothelial cells and vascular smooth muscle cells, as well as infection‐induced barrier dysfunction (as indicated by notably enhanced EB red fluorescence signals) (Figure 5D). Furthermore, SEM analysis revealed prominent endothelial damage caused by EBOV infection, as evidenced by the marked endothelial cell rounding and detachment (Figure 5E). These consistent results provide the first evidence that live EBOV infection recapitulates the vascular pathogenic effects of EGP and MGP expression, particularly in endothelial barrier disruption.

In addition, we validated the utility of this rat vascular model for evaluating therapeutic interventions targeting GP‐mediated pathogenicity and filoviral infection. As shown in Figure 5F, treatment with interferon‐beta (IFN‐β) and tunicamycin significantly attenuated the MGP‐induced increase in vascular permeability. Moreover, both interventions markedly reduced vascular leakage caused by live EBOV infection (Figure 5G). Collectively, these findings highlight the potential of this rat vascular model not only for studying hemorrhagic fever virus pathogenesis (via viral pathogenic protein expression or authentic infection) but also for testing and developing targeted antiviral therapies.

## 3 Discussion

**FIGURE 6:**
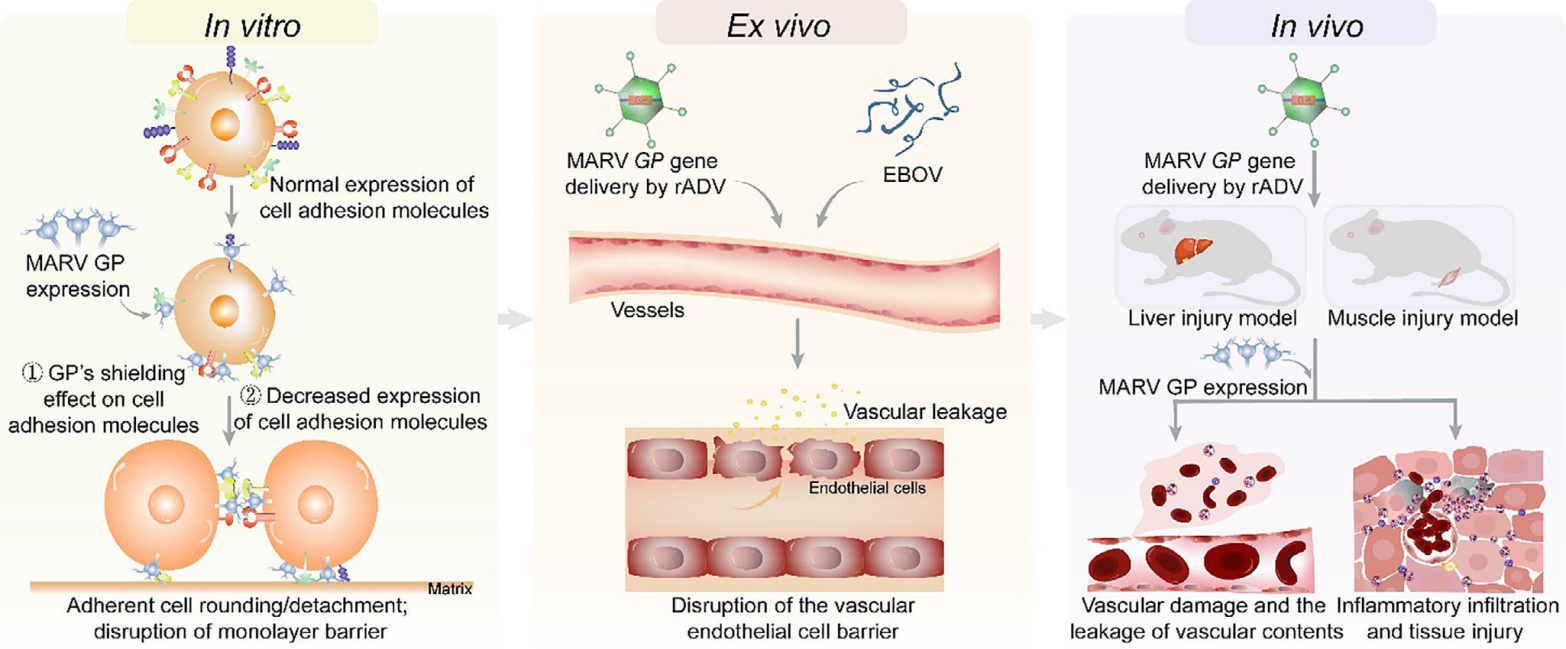
Summary of the research models used in this study. First, using in vitro models of cultured cells and cell monolayers, MARV GP (MGP) could interfere with cell surface molecules, likely through both shielding effects and downregulation of their expression, reducing cell‐cell/matrix adhesion and causing cell rounding and detachment. The effects of MGP are largely attributed to its mucin‐like domains (MLD). Using an ex vivo rat vessel model, we demonstrated that MGP disrupts the vascular endothelial barrier and leads to increased vascular permeability, and that MLD is required for this vascular pathological effect of MGP. In addition, using this vessel model, we found for the first time that EBOV infection damages the vascular endothelial barrier and induces vascular leakage, and that the vascular damage caused by both MGP and EBOV infection can be inhibited by drugs, indicating that this vessel gene transfer or infection model may also be used in intervention therapy research. Finally, using in vivo transduction models, we demonstrated the effects of MGP (but not the MLD‐deleting mutant) on increasing vascular permeability, inducing or exacerbating inflammatory infiltration, and tissue damage in the liver, a major target organ of filovirus infection, or in an in situ gastrocnemius model.

As one of the most dangerous human pathogens known, MARV poses a significant threat to public health. With increasing global communication between countries and regions, life‐threatening hemorrhagic fever caused by MARV has spread to a broader area. The potential misuse of MARV as a bioweapon has led to its designation as a CDC Category A Bioterrorism Agent [52]. However, no specific antiviral drugs or vaccines have been approved for use [53]. Moreover, viral pathogenesis, including pathogenic factors and mechanisms, remains elusive, underscoring the ongoing difficulties and challenges in antiviral drug development. In this study, we identified and characterized MGP as a critical pathogenic factor using multiple in vitro, ex vivo, and in vivo models. First, in cultured cell models, MGP interfered with cell surface molecules, likely through shielding effects and downregulation of their expression, resulting in decreased cell adhesion and disruption of the cell layer barrier. Using an ex vivo rat vessel transduction model, MGP disrupted the vascular endothelial barrier, leading to increased vascular permeability. Moreover, using ex vivo cultured rat vessels, we identified for the first time that EBOV infection, similar to GP expression, damaged the vascular endothelial barrier and increased vascular permeability. In addition, the ex vivo model established in this study was used to evaluate pharmacological interventions against the pathogenic effects of filoviral infection and GP expression. Furthermore, using in vivo liver and muscle transduction models, we confirmed that MGP increased vascular permeability and promoted inflammatory cell infiltration and tissue damage (Figure 6). In addition, deletion of the MLD significantly attenuated all GP‐associated pathogenic effects, highlighting its critical role in GP‐driven pathogenesis. Together, using these multiple models, this study uncovered the role of MGP as a notable pathogenic factor likely involved in the hemorrhagic process during MARV infection, which may help advance our understanding of MARV pathogenesis and the development of targeted therapeutics against filoviral infections or the pathogenesis of GP.

The most notable clinical feature of MARV infection is its propensity to cause hemorrhage. Disruption of epithelial and endothelial barriers may play a pivotal role in MARV pathogenesis, including hemorrhagic development. However, prior to this study, it remained completely unknown which factor of MARV impairs such cellular barriers and contributes to hemorrhage, representing a significant gap in the understanding of MARV pathogenesis that urgently required investigation. In contrast, some progress has been made in identifying potential pathogenic factors of EBOV responsible for vascular damage, particularly its full‑length GP, which has been shown to interfere with endothelial adhesion, mediate immune evasion from neutralizing antibodies, and modulate inflammatory responses [40, 54, 55]. Moreover, as noted earlier, EBOV also encodes several soluble GP isoforms—notably its shed GP, which may function as a decoy for antibodies targeting the full‑length membrane‑bound GP, thereby contributing to viral immune evasion [56]. Shed GP has also been suggested to stimulate cellular inflammation, and in vitro studies have reported that it may induce barrier disruption and enhance permeability in cultured adherent monolayers [57]. Nevertheless, MARV and EBOV belong to distinct genera (*Orthomarburgvirus* and *Orthoebolavirus*, respectively) within the family *Filoviridae*. The GP of MARV exhibits substantial sequence divergence from that of EBOV—especially in their MLDs—and differs markedly in transcriptional and post‑translational processing. Notably, MARV does not produce the soluble GP isoforms characteristic of EBOV. Furthermore, even Reston virus, which is more closely related to EBOV within the same genus, exhibits distinct pathogenicity; it is non‑pathogenic in humans, and its full‑length GP fails to disrupt—or relatively weakly affects—cell adhesion [43, 58]. Therefore, the functions of MARV proteins cannot be simply inferred from previous studies on EBOV, underscoring the need to identify the pathogenic factors underlying MARV‑induced hemorrhage. Here, by using the in vitro models of cultured adherent cells, MGP expression may not directly affect cell viability but can induce cell rounding and attachment and disrupt the barrier function of cell monolayers. This effect is linked to damage to vascular endothelial cells and an increase in vascular permeability by MGP, as observed in our subsequent experimental models. Moreover, by maintaining host cell viability to enable consistent viral replication while simultaneously disrupting cell adhesion and barrier function, this mode of action may hold more balanced physiological significance for viral infection and pathogenesis than rapid cytotoxicity. Previous studies have shown that EGP can induce morphological changes in cells, and the proposed mechanism is shielding by its glycans [41]. Although MGP and EGP share low sequence identity, MGP possesses extensive glycosylation as well, and a similar spatial shielding of cell surface molecules by MGP is likely. ITGB1 is a ubiquitous integrin subunit critical for cell adhesion and a candidate attachment factor for filovirus entry [59]. HLA‐I is constitutively expressed on nucleated cells and is central to antigen presentation and cytotoxic T lymphocyte responses, which are crucial for antiviral defense against MARV infection [60]. Thus, we assessed these molecules as representative adhesion and immune‐related surface factors relevant to pathogenesis, respectively. Indeed, our results show that full‐length MGP containing MLD significantly downregulated detectable cell surface levels of both ITGB1 and HLA‐I. Furthermore, treatment with the glycosylation inhibitor tunicamycin significantly reversed MGP‐mediated cell rounding, detachment, and shielding of the cell‐adhesion protein ITGB1, supporting the potential spatial shielding by the extensive glycosylation of MGP, especially in its MLD. Further mutagenesis and deletion analyses revealed that the N‐terminal region of the MLD, in particular, appears to be more critical for the anti‐adhesive function of GP, whereas the abolition of some individual glycosylation sites had no significant impact. Additional mutational studies targeting the signal peptide or transmembrane domain of GP—which could mimic the shed GP form observed in EBOV—highlighted the importance of its membrane localization for activity, although the full‐length protein context may also be required. Besides, even the incoming full‐length MGP displayed on the membrane of virions (pseudovirions) failed to induce obvious cell morphological changes (Figure S5), further suggesting that the pathogenic effect likely depends on the form of MGP expressed on the plasma membrane of transfected or infected cells. Collectively, these data support the important mechanism whereby MGP, through its plasma membrane expression and the dense glycosylation of its MLD, mediates a spatial shielding effect on cell surface molecules, leading to the deadhesion. In addition, the GP of EBOV, as well as those of other filoviruses (except MARV), may harbor alternative glycosylation modifications—such as C‐mannosylation [58]. These molecular disparities, together with other differences in encoding strategies and post‐translational processing likely exert impacts beyond direct viral life cycle processes (including virion packaging, intracellular trafficking, and host cell entry) and may also significantly modulate host immune recognition, inflammatory responses, and overall pathogenicity. A deeper mechanistic understanding of how these differences contribute to distinct infection pathogenesis remains an important and compelling direction for future research.

Intriguingly, our further analyses, including transcriptome profiling, suggest that MGP may also induce the global downregulation of adhesion‐related molecule expression, which appears to be largely attributed to MLD. This is likely an additional mechanism underlying the cytopathic effects of MGP, which has not yet been reported for EGP. It would be interesting to further investigate how MGP and MLD downregulate the expression of these molecules. Concurrently, transcriptome mining indicates that genes associated with endoplasmic reticulum stress and the unfolded protein response also seem to be differentially regulated by MGP, suggesting that its functional roles are multifaceted and complex. Further analyses in HUVECs also confirm and expand that MGP exhibits diverse regulatory effects: it suppresses the expression of various surface adhesion factors and immune molecules (e.g., HLA and IFN‐β1); it downregulates anti‐inflammatory IL‐10, while upregulating multiple inflammatory factors. Thus, the pathogenicity of MGP likely results from the integrated effects of multiple mechanisms identified in this study, including its high glycosylation status—particularly within the MLD region—which may confer steric shielding on the cell surface; transcriptional repression of adhesion molecules; induction of ER stress/UPR pathways with potential disruption to endothelial cell activity and adhesion molecule processing; promotion of inflammation; and suppression of both innate and adaptive immune responses. These findings and follow‐up investigations in the future may help address a comprehensive understanding of MARV pathogenesis.

Among the complex factors causing hemorrhage, impairment of vascular integrity, particularly the loss of endothelial layer integrity and barrier function, likely serves as a direct and critical contributor. Injury to vascular endothelial cells severely impairs vessel function, increases vascular permeability, and promotes the infiltration of inflammatory cells, which then affects the stability of blood platelets and other components. In addition, endothelial cell injury can cause fibrin exudation, which may further exacerbate disseminated intravascular coagulation (DIC). These effects may ultimately contribute to bleeding [23]. Therefore, damage to blood vessels, particularly endothelial cells, likely plays a significant role in the hemorrhagic manifestations associated with filovirus infections. In this study, using an ex vivo model, MGP directly disrupts the barrier function of blood vessels and significantly increases vascular permeability. Therefore, MGP may be an important direct factor leading to hemorrhagic symptoms. Furthermore, it is conceivable that the pathogenic effect of MGP damages the integrity of the blood‐brain barrier, blood‐retinal barrier, and other vascular barriers. Consequently, it may contribute to other significant clinical symptoms resulting from viral infections and act as a potential key factor involved in viral pathogenesis.

In addition, we established a rat vascular model to analyze MGP's pathogenicity in vessels. The rat vascular model offers significantly higher accessibility and cost‐effectiveness than larger blood vessels from humans or nonhuman primates. By leveraging recombinant adenoviral vectors for gene delivery, this ex vivo rat vascular transduction model provides a simpler and more economical platform for studying the pathogenic determinants and mechanisms of highly virulent viruses that can be operated in laboratories with lower biosafety levels. Moreover, using our rat vascular model, we demonstrated that authentic EBOV (tested here because of the absence of live MARV in our biosafety laboratory) could infect vascular tissues, disrupt endothelial barrier integrity, and induce vascular leakage. This is the first experimental evidence confirming the vascular infectivity and pathogenicity of authentic filoviruses. Finally, we used this rat ex vivo vascular model, combined with rADV‐mediated MGP expression or live EBOV infection, to evaluate the interventional effects of IFN‐β (suppressing viral infection) and tunicamycin (inhibiting glycosylation), highlighting the utility and potential of these models as tools for evaluating therapeutic interventions. However, this model has several limitations. Isolated vessels may experience natural physiological damage during in vitro culture, which can restrict both the culture and infection processes of the pathogen. Furthermore, given that endothelial cells form only a single layer attached to the inner vessel wall, they are particularly vulnerable to damage during culture and preparation. Therefore, meticulous handling was required throughout the experiments. These factors indicate the need for further refinement in future studies.

The liver is the primary target organ for filovirus infection. Liver damage caused by complex direct and indirect factors associated with viral infections is closely related to coagulation dysfunction, DIC, bleeding, and immune/inflammatory disorders, significantly contributing to the pathogenesis of viral hemorrhagic fever [48]. Using our in vivo liver transduction model, we confirmed the increase in vascular permeability caused by MGP and MLD. Moreover, histopathological analysis revealed that MGP‐induced vascular congestion and red blood cell leakage (hemorrhage) and exacerbated inflammatory cell infiltration and hepatocyte damage. Similar pathological changes have been reported in animal models infected with authentic MARV [61]. These effects may further aggravate increased vascular permeability, inflammatory disorders, and tissue damage, facilitating the progression of hemorrhagic fever. In addition, we used a muscle transduction model established previously [39], which enables convenient in‐situ viral vector transduction, as well as monitoring and analysis of gene expression and pathogenicity, to further verify the pathogenic effects of MGP, including damage to the vascular system (inducing congestion and bleeding), enhanced inflammatory infiltration, and tissue damage. Besides damaging a variety of tissues and organs, such as the liver, spleen, lymph nodes, endocardium, and reticuloendothelial system, MARV infection can also lead to muscular tissue injury in patients [22]. However, muscle damage may not be the primary cause of high viral lethality. Therefore, this local muscle injury model still primarily serves to corroborate and further demonstrate the pathogenic effects of MGP. Additionally, owing to its convenience in both technical manipulation and phenotypic observation, it can offer a practical and complementary in vivo platform for investigating the pathogenic factors of hemorrhagic fever viruses and for evaluating potential therapeutic interventions. Together, the data from these in vivo models validated the increased vascular permeability and the induction of inflammatory infiltration and tissue damage mediated by MGP, further revealing MGP's pathogenic effects.

In addition to the technical challenges mentioned earlier, such as the relatively high operational demands of the ex vivo vessel model, this study has several other limitations or noteworthy aspects that warrant follow‐up investigations in the future. In this study, we utilized authentic EBOV for the first time to analyze the direct infectivity and pathogenicity of filoviruses toward blood vessels. As extensively discussed above, although EBOV and MARV both belong to the *Filoviridae* family and share many similarities in molecular biology and clinical pathology, they still exhibit substantial differences. Moreover, even our artificially constructed GP lacking the transmembrane domain did not exhibit the pathogenic effects associated with the corresponding EBOV protein, shed GP, further suggesting potentially important functional differences between the two viral GPs. In summary, the differences in their pathogenicity and other biological functions (such as entry, assembly, and immune evasion), and the consequent impact on viral pathogenesis, still require further investigation. While this study provides many potentially useful clues for comparing and deciphering the pathogenic mechanisms of these two most notorious hemorrhagic fever viruses, the effects of live MARV infection on blood vessels remain to be further experimentally tested. Furthermore, the effects on cellular status during infection are likely to be more complex, and the expression context of GP involves differences. Particularly, co‐expression of other viral proteins may synergistically contribute to viral pathogenesis. Therefore, the role of MARV GP as a hemorrhagic pathogenic factor identified in this study warrants further analysis with more experiments of live virus infections in future studies. Moreover, our analyses suggest that the pathogenicity of MARV GP itself may result from multiple converging mechanisms, such as the proposed steric shielding of cell adhesion via the MLD‐mediated “glycan shield” at the cell surface, global downregulation of cell surface molecules, suppression of innate and adaptive immune responses, promotion of inflammatory responses, and potential modulation of pathways such as ER stress/UPR. These multifaceted effects may interact with or potentiate one another, collectively contributing to MGP pathogenicity. However, the interplay or causal relationships among these effects, as well as their underlying mechanisms, seems to be complex. For example, while MGP appears to enhance the expression of certain key pro‐inflammatory genes while suppressing the anti‐inflammatory cytokine IL‐10, the mechanisms by which MGP differentially regulates inflammatory and anti‐inflammatory gene expression have not been further elucidated herein. The causality involving ER stress/UPR pathway regulation also requires further investigation through additional pathway intervention or rescue experiments. Additionally, this study reports the impact of MGP expression on the cellular transcriptome; however, in more complex systems—particularly in vivo models—MGP may exert even more intricate effects on tissue and organ transcriptomes. Furthermore, other physiologically relevant systems (such as more types of endothelial barriers, iPSC‐derived endothelial systems, vascular organoids, liver organoids, and lymphoid or macrophage systems) could be employed in future studies to test the newly identified effects of MGP reported herein. Therefore, it is important to note that in vitro and ex vivo models may only partially recapitulate the effects of MGP; in vivo systems are likely more complex, and findings from these various systems should be integrated and interpreted collectively. Although this study provides research models ranging from cell monolayer barriers to ex vivo murine vessels and in situ organ models—offering useful tools for identifying viral pathogenic factors and mechanisms, as well as for intervention development—the artificial nature of these models should also be noted. In the future, closer integration with clinical contexts will be necessary to interpret the findings in a way that yields greater clinical value. Lastly, how EGP affects the host cell transcriptome has not yet been reported and remains to be investigated—such studies would further help elucidate the unique and shared features and mechanisms of pathogenicity among different viruses, thereby facilitating the development of both specific and broad‐spectrum interventions against hemorrhagic fever viruses.

In summary, this study uncovered the role of MARV GP as a critical pathogenic factor that damages endothelial cells and vascular integrity and exacerbates inflammation and tissue injury using multiple in vitro, ex vivo, and in vivo models. Moreover, using an ex vivo rat vascular model, we demonstrated the pathogenicity of authentic filovirus (EBOV) infection and the utility of the model as a tool for intervention research against viral, particularly GPs’ pathogenicity in vessels. Collectively, these findings advance our understanding of filovirus pathogenesis and provide potential therapeutic targets and practical experimental models. The established models and methodologies may also be applicable to studies on the pathogenic factors, mechanisms, and therapeutic developments of other highly pathogenic HFVs.

## 4 Materials and Methods

### 4.1 Ethics Statement

All animal experiments were performed in accordance with the animal ethics guidelines of the Chinese National Health and Medical Research and were approved by the Animal Care and Use Committee and Ethical Committee of the Wuhan Institute of Virology, Chinese Academy of Sciences (permit numbers: WIVA23202103 and WIVA23202201).

### 4.2 Cell Lines, Plasmids, and Viruses

Human embryonic kidney 293T (HEK293T; American Type Culture Collection [ATCC]; RRID: CVCL_0063) and human embryonic kidney 293A cells (HEK293A; National Virus Resource Center [NVRC]; CSTR: 16533.09. IVCAS09.0216), and human umbilical vein endothelial cells (HUVEC; NVRC; CSTR: 16533.09. IVCAS09.0227), human hepatocarcinoma (Huh7, NVRC; CSTR: 16533.09. IVCAS09.0238) and SW13 cells (ATCC; RRID: CVCL_0542) were grown in Dulbecco's modified Eagle's medium (DMEM) supplemented with 10% fetal bovine serum (FBS; Gibco, USA). Human embryonic kidney (HEK293, ATCC; RRID: CVCL_0045), human cervical adenocarcinoma (HeLa, ATCC; RRID: CVCL_0030), and African green monkey kidney (Vero, ATCC; RRID: CVCL_0059) cells were maintained in modified Eagle medium (MEM) with 10% fetal bovine serum. All cell lines were routinely tested for Mycoplasma contamination and were grown at 37°C in a 5% CO_2_ incubator.

MARV GP was derived from the Lake Victoria Marburg virus strain (Lake Victoria Marburg virus strain Uganda 371Bat2007; GenBank accession number: FJ750958). Full‐length MGP was obtained by gene synthesis (BGI, China), and the MLD (amino acids 768–1526) was removed to obtain MGPΔMLD. For efficient eukaryotic protein expression, MGP and MGPΔMLD were cloned into pCAGGSP7, with an S‐tag fused to the C‐terminus for convenient detection. Other related truncations or mutations are constructed in a similar manner. All plasmids were constructed using standard molecular biology techniques.

To generate recombinant adenoviruses, MGP and MGPΔMLD, as well as EGP and EGPΔMLD were cloned into the expression plasmid pAdTrack‐CMV of the adenovirus generation system, which contains an EGFP expression cassette as a reporter for convenient monitoring of gene expression. Subsequent homologous recombination with the pAdEasy skeleton plasmid and the generation, amplification, and purification of recombinant adenoviruses were performed as previously described [39].

To generate the MGP/MGPΔMLD pseudotyped viruses, HEK293T cells transfected with MGP or MGPΔMLD expression plasmids were infected at 12 h post‐transfection with VSVΔG/GFP‐VG in which the *G* gene of VSV is replaced with GFP [62]. After 2 h of adsorption at 37°C, the viruses were removed, and the cells were extensively washed three times with serum‐free DMEM. After 24 h of incubation at 37°C in DMEM supplemented with FBS, the culture supernatants were centrifuged and filtered through a 0.45‐µm filter to remove cell debris, followed by storage at −80°C until further experimentation.

The Ebola virus Zaire strain (Zaire/1976/Mayinga; GenBank accession number: AF086833.2) was obtained from the NVRC (CSTR: 16533.06. IVCAS6.7478) and propagated in Vero cells. Experiments on live EBOV infection were performed in a Biosafety Level 4 (BSL‐4) laboratory, Wuhan Institute of Virology, Chinese Academy of Sciences. Viral titers were determined using the 50% tissue culture infectious dose (TCID50) assay.

### 4.3 Counting of Detachment Cells and Cell Viability Detection

HEK293T cells were seeded in 24‐well plates at a density of 1 × 10^5^ cells/well and co‐transfected with MGP or MGPΔMLD expression plasmids along with pEGFP‐N1. After 24 h, the cells were photographed using a fluorescence microscope (EVOS M5000; Thermo Fisher Scientific, USA). Detached cells in the supernatant and adherent cells (following trypsin digestion) were collected and counted (Countstar, China) to determine the cell detachment rate. To investigate the effects of glycosylation inhibition, transfected HEK293T cells were treated with tunicamycin (2 µg/mL; #ab120296, Abcam) for 24 h before subsequent analyses.

To assess cell viability, HEK293T cells were seeded in 96‐well plates at a density of 1 × 10^4^ cells/well and transfected with expression plasmids. At 24 h post‐transfection, cell viability was measured using the Cell Counting Kit‐8 assay (#C0038, Beyotime).

To evaluate cell re‐adhesion ability, detached cells were collected from the culture medium 24 h post‐transfection, while adherent cells were harvested by trypsinization. Equal numbers of cells from each population were seeded into new culture plates and maintained under standard conditions. Cell adherence rates were quantified at multiple time points, and cell viability was assessed using the trypan blue (#C0011, Beyotime) exclusion method.

### 4.4 Western Blotting (WB) and Immunofluorescence Assay (IFA)

Cell samples were incubated with the lysis buffer (pH 7.4, 150 mm NaCl, 1 mM EDTA, 1% Triton X‐100) supplemented with a cocktail of protease inhibitor (#04693132001, Roche) at 4°C for 30 min [62, 63]. Following centrifugation (13000 × *g*) at 4°C for 10 min, the supernatants of the cell lysates were mixed with the sodium dodecyl sulfate (SDS) sample buffer (25% glycerol, 2.5% SDS, 125 mM Tris, pH 6.8, 125 mM dithiothreitol, 0.25% bromophenol blue) and boiled for 10 min. Proteins were then separated by SDS‐PAGE and transferred to polyvinylidene difluoride (PVDF) membranes (#L3000015; Millipore). After blocking with 5% non‐fat milk in TBST (Tris‐buffered saline with 0.1% Tween20, pH 7.4), the membranes were incubated with primary antibodies against the S‐tag (1:3000; #ab101290‐T38, Sino Biological) and β‐actin (1:5000; #20536‐1‐AP, Proteintech), followed by the corresponding HRP‐conjugated secondary antibodies (1:10000; #SA00001‐2, Proteintech). The membranes were washed extensively with TBST between each step. Protein signals were detected using an enhanced chemiluminescence (ECL) kit (#34577; Thermo Fisher Scientific).

For IFA to analyze subcellular protein localization, transfected or transduced cells were fixed with 4% paraformaldehyde in phosphate‐buffered saline (PBS) and permeabilized with 0.5% Triton X‐100‐PBS on ice for 15 min [39, 64, 65, 66, 67]. After blocking with 5% bovine serum albumin (BSA; #BS114, Biosharp), the cells were incubated overnight at 4°C with primary antibodies against the S‐tag (1:1000) or MARV GP (1:1000). The cells were then incubated with Alexa Fluor 647‐labeled secondary antibodies (1:1000; #ab150079; Abcam) for 1 h at RT. To visualize the nuclei, the cells were incubated with Hoechst 33258 (#C1011, Beyotime) for 5 min at RT. Images were acquired and analyzed using a laser confocal microscope (STELLARIS 8; Leica Microsystems, Wetzlar, Germany).

### 4.5 Cell Monolayer Permeability Detection with RTCA

For real‐time cell analysis, 100 µL of medium was first added to an E‐plate 96 (Agilent, USA) to measure the background impedance. HEK293T cells were resuspended in the medium and seeded into E‐plate 96 at a density of 1 × 10^4^ cells/well. After incubation at RT for 30 min, the E‐plate was placed on an RTCA test table in a cell incubator. An RTCA SP instrument (Agilent Technologies) was used to automatically monitor cell adhesion, spreading, and proliferation every 15 min. Approximately 18 h post‐seeding, the cells were transfected with MGP, MGPΔMLD expression plasmids, or a control vector and monitored for 60 h. The measured impedance signals from each well were automatically converted into cell index (CI) values using the RTCA software, and the curves of the cell monolayer barrier index values over different time periods were obtained.

### 4.6 Flow Cytometry Assay

To assess the cell surface signals of ITGB1 and HLA‐I, HEK293T cells were co‐transfected with MGP or MGPΔMLD expression plasmids and pEGFP‐N1. At 24 h post‐transfection, the cells were digested with trypsin and fixed with 4% paraformaldehyde. After blocking with 5% BSA in PBS for 1 h at RT, the cells were incubated with PE‐conjugated anti‐human ITGB1 antibody (5 µL/1 × 10^6^ cells; #12‐0299‐42, eBioscienc) or PE‐conjugated anti‐human HLA‐I antibody (5 µL/1 × 10^6^ cells; #311406, BioLegend) at RT for 20 min. ITGB1 and HLA‐I expression on the cell surface was determined by flow cytometry (FACSAria III, Becton, USA) and analyzed using FlowJo software. For each sample, 10 000 events within the live cell gate (determined by forward and side scatter) were analyzed. Negative and positive gates for EGFP and PE were established based on the negative controls, and scatter plots correlating the EGFP and PE fluorescence signals were generated. To further assess the detachability of ITGB1 and HLA‐I signal, the mean fluorescence intensity (MFI) of PE in the EGFP‐positive population or the total live cell population was analyzed. To assess the total expression of intracellular and cell surface ITGB1 proteins, cells were fixed with 4% paraformaldehyde and permeabilized with 0.5% Triton X‐100‐PBS before IFA and flow cytometry.

### 4.7 RNA Extraction and Quantitative Real‐Time qPCR

Total RNA was extracted using TRIzol reagent (#15596018, Invitrogen) and reverse transcribed using the PrimeScript RT Reagent Kit with gDNA Eraser (#047A, Takara). Quantitative real‐time PCR (qRT‐PCR) was performed using SYBR Premix Ex Taq II (#820A, Takara) on a StepOnePlus Real‐Time PCR System (Applied Biosystems, USA). Relative quantitation was performed using the 2^−ΔΔCT^ method, with GAPDH as an internal control, and the relative fold change was calculated by normalizing to the control. The sequences of the gene‐specific primer pairs are listed in Table S1.

To determine the viral loads of EBOV‐infected vessels, total RNA was extracted from the vessels 48 h post‐infection using TRIzol reagent after homogenization. One‐step qRT‐PCR was then performed using the One‐Step PrimeScript RT‐PCR Kit (#RR064A, Takara) and a StepOnePlus Real‐Time PCR System. The primers and probes used are described in Table S1.

### 4.8 Transcriptome Analysis

HEK293T cells were transfected with MGP or MGPΔMLD expression plasmids and the control vector for 24 h. Total RNA was extracted and shipped to BGI on dry ice for library preparation and Illumina sequencing. The sequence reads were aligned to the human genome reference sequence (GCF_000001405.39_GRCh38.p13). The integrated quality control for the expressed genes was set to a false discovery rate (FDR) of less than 0.05. To visualize the differentially expressed genes (DEGs) among the groups, we identified and analyzed the DEGs across the three groups. The threshold absolute value of the log fold change (logFC) was set to 0.5, and the p‐value was set to 0.05, indicating a significant difference. The results were visualized using R and RStudio software. For Gene Ontology (GO) and Kyoto Encyclopedia of Genes and Genomes (KEGG) analyses, the ID lists of DEGs were mapped to their corresponding GO or KEGG terms. GO terms were classified based on their roles in cellular components (CC), molecular function (MF), and biological processes (BP). Enrichment analysis was performed using R packages, and multiple testing corrections were conducted using the Benjamini‐Hochberg procedure to control the FDR, with a threshold value of 0.05. The results were visualized using R and RStudio to represent the enriched KEGG pathways or GO terms and their relationships. To analyze adhesion‐related molecules or the genes related to ER stress/UPR, the expression levels of these molecules were measured in each sample. Log_2_ transformation was performed to stabilize the variance, and a heatmap was generated using the R and R Studio visualization tools. The RNA‐Seq datasets generated in this study have been deposited in the Science Data Bank (CSTR: 31253.11. sciencedb.28198;https://www.scidb.cn/s/ArIJZn).

### 4.9 Scanning Electron Microscopy Analysis

For SEM, glutaraldehyde‐fixed vessels were rinsed with PBS and dehydrated using alcohol gradients. After drying using a critical‐point dryer (EM CPD300, Leica, Germany), the samples underwent conductive treatment using a sputter coater (EM ACE200, Leica, Germany) and were imaged using a scanning electron microscope (SU8010; Hitachi, Japan).

### 4.10 Construction of Vascular Injury Model ex vivo

To obtain vessels for subsequent culture and infection, 6‐8‐week‐old female Wistar rats (Charles River) were euthanized by CO_2_ inhalation and exposure to the thoracic cavity. A small incision was made in the right atrium for drainage. The vasculature was perfused via the left ventricle with ice‐cold phosphate‐buffered saline (PBS) or saline until the effluent was cleared and the liver appeared bloodless. The heart, lungs, and surrounding tissues were then dissected to expose the aorta, which was excised, cleaned of the surrounding fat, and cultured in RPMI 1640 supplemented with 10% FBS, penicillin, and streptomycin at 37°C in a 5% CO_2_ incubator. All procedures were performed aseptically.

For recombinant adenovirus transduction, the isolated vessels were trimmed to approximately 3 cm and cultured in 6‐well plates within 6 h of isolation. Purified recombinant adenovirus (1 mL) at a titer of 1 × 10^8^ TCID50/mL was added to cover the vessel. To enhance the transduction of cells inside the vascular lumen, the adenovirus was simultaneously injected into the lumen. After incubation for 1.5 h, the recombinant adenovirus was discarded, and 2 mL of fresh medium was added for further culture. After 48 h, the vessels were washed with PBS, fixed with glutaraldehyde, and prepared for SEM analysis.

To detect vascular permeability, the vessels were ligated at both ends, and 0.02% Evans Blue (EB; #E6135, Macklin) was injected into the vascular lumen for 2 min to achieve complete staining. Following this, the EB in the lumen was washed with PBS, and the vessels were fixed with 4% paraformaldehyde for photography and subsequent pathological analysis.

For EBOV infection, the vessels were cut into approximately 1 cm segments and placed in a 24‐well plate. Subsequently, 1 mL of EBOV at a titer of 1 × 10^7^ TCID50/mL was added and incubated for 1 h. After incubation, the virus was discarded, and RPMI 1640 medium was added for an additional 48 h of culture. For SEM imaging, the vessels were fixed with glutaraldehyde for subsequent procedures. To assess vascular permeability, the vessels were covered with 0.005% EB for 5 min. The EB was discarded, and the vessels were washed with PBS before fixation with paraformaldehyde for photography and subsequent pathological analyses.

### 4.11 Pharmacological Treatment Evaluation

Vessels were pre‐treated with 500 µg/mL of interferon‐beta (IFN‐β; #300‐02BC, Peprotech) or 8 µg/mL tunicamycin for 1 h. Subsequently, 1 mL of rADV‐MGP with a titer of 1 × 10^8^ TCID50/mL or EBOV with a titer of 1 × 10^7^ TCID50/mL was added and incubated for 1 h, after which the virus was removed. The culture was then continued in a medium supplemented with the respective pretreatment substances for an additional 48 h. Permeability assays were conducted as previously described, followed by EB quantification.

### 4.12 Mouse Liver and Muscle in vivo Transduction Models

To construct a mouse liver injury model, 6‐8‐week‐old female BALB/c mice (*n* = 6; Charles River) were injected via the caudal vein with 100 µL of purified recombinant adenoviral vectors with a titer of 1 × 10^10^ TCID50/mL to ensure sufficient protein expression in the liver. After 72 h, the mice were euthanized, and their livers were dissected, fixed with paraformaldehyde, and embedded in paraffin for pathological analysis.

In the hepatic vascular permeability model, 6‐8‐week‐old female BALB/c mice (*n* = 6; Charles River) received an intravenous injection of EB (40 mg/kg) 72 h after transduction with 100 µL of 2 × 10^10^ TCID50/mL recombinant adenoviruses. After 1 h of systemic circulation, the mice were euthanized, and the remaining EB in the blood vessels was washed away with PBS through cardiac perfusion. The livers were harvested to quantitatively analyze EB and fixed with paraformaldehyde for subsequent pathological analyses.

For the muscle injury model, 6‐8‐week‐old female BALB/c mice (*n* = 7; Charles River) were injected with 50 µL of 1 × 10^10^ TCID50/mL purified recombinant viral vectors into the gastrocnemius of the hind limb. At 72 h post‐transduction, the mice were anesthetized via intraperitoneal injection of Avertin (200 µL/10 g) for live imaging. EGFP expression was monitored by fluorescence imaging as an indicator of recombinant adenoviral transduction. The images were analyzed by spectral separation using the IVIS Spectrum In Vivo Imaging System (PerkinElmer Inc., USA; Living Image 4.3.1 software). The relative intensities of the emitted light are presented as the average radiance in p/s/cm^2^/sr and displayed using an RGB color representation. After euthanasia, the injected gastrocnemius muscle was dissected and fixed in paraformaldehyde for histopathological examination.

### 4.13 Quantitative Analysis of Evans Blue

To quantify the EB intake, stained vessels or livers were weighed and incubated in 1 mL of formamide (#F810079, Macklin) for 48 h at 60°C to extract the EB. Following extraction, the samples were centrifuged at 5 000 × *g* for 5 min, and the absorbance of the supernatant was measured at 620 nm using a spectrophotometer. The concentration of EB in the extracts was calculated using a standard curve of EB in formamide and subsequently normalized to the corresponding tissue weight.

### 4.14 Pathological Analysis

After fixation in 4% paraformaldehyde, the tissues were dehydrated in graded alcohol solutions to remove water and cleared with xylene to facilitate paraffin infiltration. The tissues were embedded in molten paraffin wax and solidified to form blocks that supported the tissue structure. Serial sections (3 µm thick) of the tissue blocks were obtained and dried for 2 h at 55°C before follow‐up detection.

H&E staining was performed using an H&E staining kit (#G1005, Servicebio), followed by imaging using a light microscope (3D HISTECH Pannoramic MIDI, Hungary). To quantify the histopathological changes, six unduplicated visual fields of the muscle or connective tissue were selected for each sample. The degrees of pathological changes compared to those in mock‐infected samples were scored as follows: 0 = normal; 1 = minimal change (minute quantity of deformation or inflammatory cells); 2 = mild change (small quantity of deformation or inflammatory cells); 3 = moderate change (modest quantity of deformation or inflammatory cell infiltration); 4 = marked change (large numbers of deformation or inflammatory cell infiltration); and 5 = severe change (diffuse deformation or inflammatory cell infiltration; almost no remaining normal tissue). Statistical analyses were performed using the GraphPad Prism software.

For IFA staining, paraffin sections were subjected to microwave irradiation for antigen retrieval and blocked with 5% BSA for 2 h at RT. The sections were then incubated with primary rabbit anti‐EGFP antibody (1:3000) or anti‐EBOV‐VP40 antibody (1:1000; #40446‐T62, Sino Biological) overnight at 4°C. After washing, the sections were incubated with the secondary antibody, Alexa Fluor 488‐conjugated goat anti‐rabbit IgG (#ab150077, Abcam), for 2 h at RT, followed by staining of the nuclei with Hoechst 33258. The sections were imaged using a laser confocal microscope (STELLARIS 8; Leica).

### 4.15 Statistical Analysis

Statistical analyses were performed using GraphPad Prism (version 8.3.0, GraphPad Software, USA). All data are presented as mean ± SD of n biological replicates. Student's t‐test was used for comparisons between the two groups, and one‐way analysis of variance (ANOVA) was used for multiple comparisons. Statistical significance was set at *p <* 0.05. The significance levels are indicated as follows: ^****^ *p <* 0.0001, ^***^ *p <* 0.001, ^**^ *p <* 0.01, ^*^ *p <* 0.05, ns = not significant.

## Author Contributions

H.L.W. and Y.J.N. supervised the project and secured funding. Y.J.N. conceptualized and designed the study. Y.J.N. and T.Y. designed experiments. T.Y. performed most of the experimental investigations and data collection, with assistance from W.D. and J.W.S. H.L., Y.F.Y., C.S., and Z.M.Y. conducted and supported the investigations at a high‐level biosafety laboratory. T.Y., Y.J.N., H.L.W., F.D., and A.A. analyzed the data. H.L.W., Y.J.N., and F.D. provided research resources and platforms. Y.J.N. and T.Y. drafted the manuscript. Y.J.N. revised and edited the manuscript. All authors have reviewed and approved the final manuscript.

## Funding

This work was supported by the CAS Project for Young Scientists in Basic Research (YSBR‐133), Class A Youth Project of Hubei Province, National Natural Science Foundation of China (32170171), National Key Research and Development Program of China (2022YFC2303300), and the Youth Innovation Promotion Association of the Chinese Academy of Sciences (2020333), to Y.J N. The funding agencies had no role in the study design, data collection, analysis, interpretation, or writing of this manuscript.

## Conflicts of Interest

The authors declare no conflict of interest.

## Supporting information




**Supporting File**: advs75288‐sup‐0001‐SuppMat.docx.

## Data Availability

The data that support the findings of this study are available from the corresponding author upon reasonable request.
