# Supplementary material for: Marburg Virus Glycoprotein Is a Remarkable Virulent Factor Linked to Hemorrhagic Pathology: Evidence from Multimodal Experimental Systems
Source: Adv Sci (Weinh). 2026 Apr 24;13(39):e11575. doi: 10.1002/advs.202511575 (PMC13335069; doi:10.1002/advs.202511575)
Supplement: Supplementary file 1 — Supporting File: advs75288‐sup‐0001‐SuppMat.docx. [file ADVS-13-e11575-s001.docx]

**Marburg Virus Glycoprotein Is a Remarkable Virulent Factor Linked to Hemorrhagic Pathology: Evidence from Multimodal Experimental Systems**

Ting Yao^a,b,d^, Hang Liu^a,c^, Yanfeng Yao^a,c^, Wei Deng^a,b,d^, Jiawen Sun^a,b,d^, Zhenyu Kang^a,d^, Ashaq Ali ^e^, Chao Shan^a,c^, Zhiming Yuan^a,c^, Fei Deng^a,b,c^, Hualin Wang^a,b,c*^, Yun-Jia Ning^a,b,c,f*^

^a^ State Key Laboratory of Virology and Biosafety, Wuhan Institute of Virology, Chinese Academy of Sciences, Wuhan 430071/430207, China

^b^ National Virus Resource Center, Wuhan Institute of Virology, Chinese Academy of Sciences, Wuhan 430071/430207, China

^c^ State Key Laboratory of Virology and Center for Biosafety Mega-Science, Chinese Academy of Sciences, Wuhan 430071/430207, China

^d^ University of Chinese Academy of Sciences, Beijing 101408, China

^e^ Center of Excellence in Science and Applied Technologies, Islamabad 75000, Pakistan

^f^ Hubei Jiangxia Laboratory, Wuhan 430200, China

*** Correspondence:**

Prof. Dr. Hualin Wang, Wuhan Institute of Virology, Chinese Academy of Sciences, Wuhan 430071/430207, P.R. China. E-mail: h.wang@wh.iov.cn

Prof. Dr. Yun-Jia Ning, Wuhan Institute of Virology, Chinese Academy of Sciences, Wuhan 430071/430207, P.R. China. nyj@wh.iov.cn

**Keywords:** Marburg virus (MARV), filovirus, viral hemorrhagic fever, Ebola virus (EBOV), glycoprotein (GP), mucin-like domain (MLD), vascular permeability, research models.

**Supplementary data**


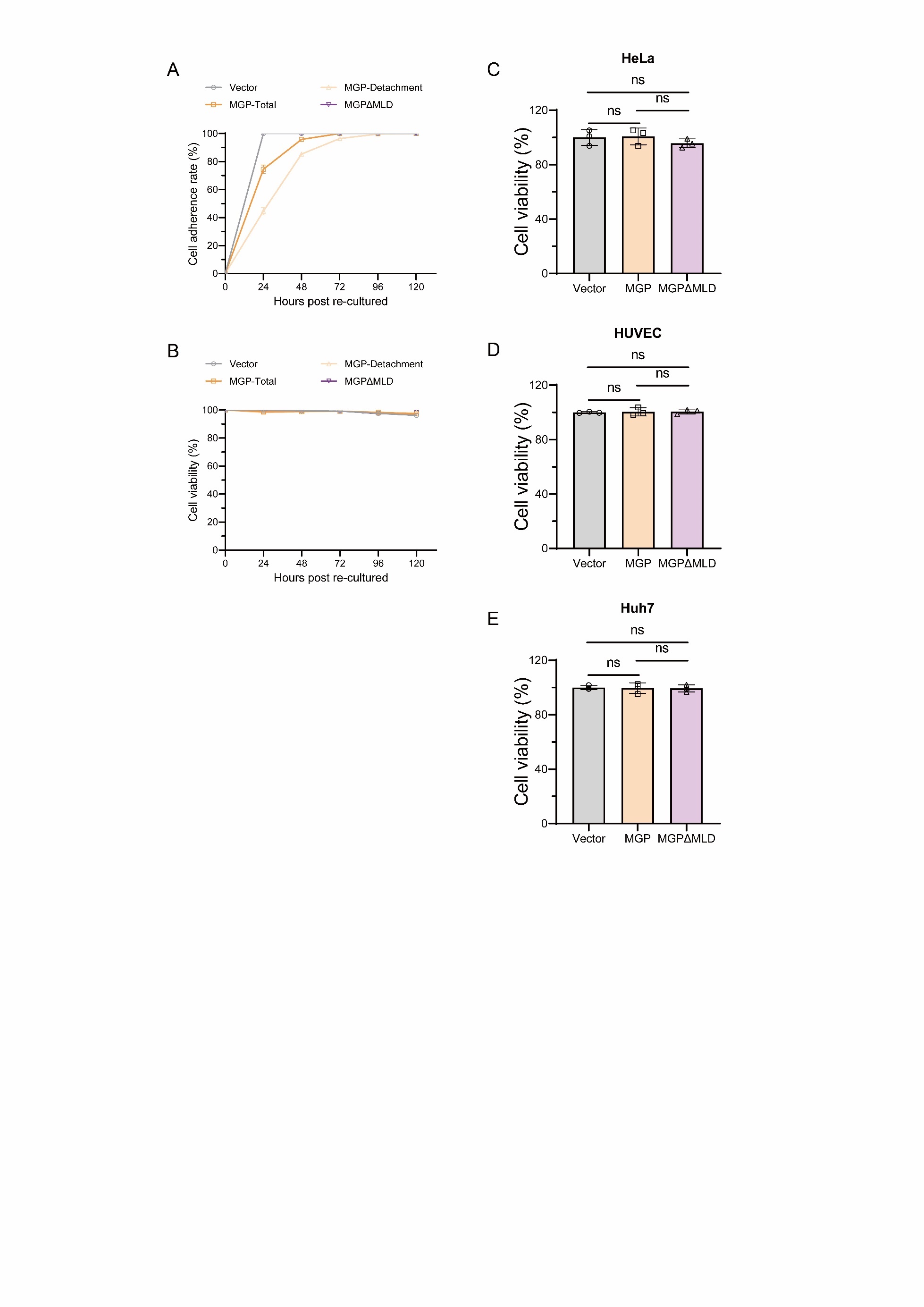


**Supplementary Figure S1. Effects of MGP and MGPΔMLD transient expression on cell viability and re-adhesion.**

**A** and **B**. HEK293T cells were transfected with plasmids expressing full-length MGP, MGPΔMLD, or a control vector. After 24 h, the total cells from the three transfection groups (designated MGP-Total, Vector, and MGPΔMLD populations), along with the detached cells from an additional MGP transfection group (designated MGP-Detachment), were harvested, respectively. Each of these populations was plated into separate 24-well plates at an equal cell density for further cultivation. At serial time points post-plating, the proportion of cells that had attached to the well surface was calculated as the cell adherence rate (**A**), and cell viability was also assessed using the trypan blue exclusion assay (**B**). Each curve represents the mean and SD of three independent biological replicates. **C-E**. HeLa, HUVEC, and Huh7 cells were respectively transfected with the indicated expression or control plasmids. Cell viability was analyzed 24 hours post-transfection using a CCK-8 assay. Data are presented as mean ± SD, n = 3 biological replicates. One-way analysis of variance (ANOVA) was used for multiple comparisons. ns, nonsignificant.

**
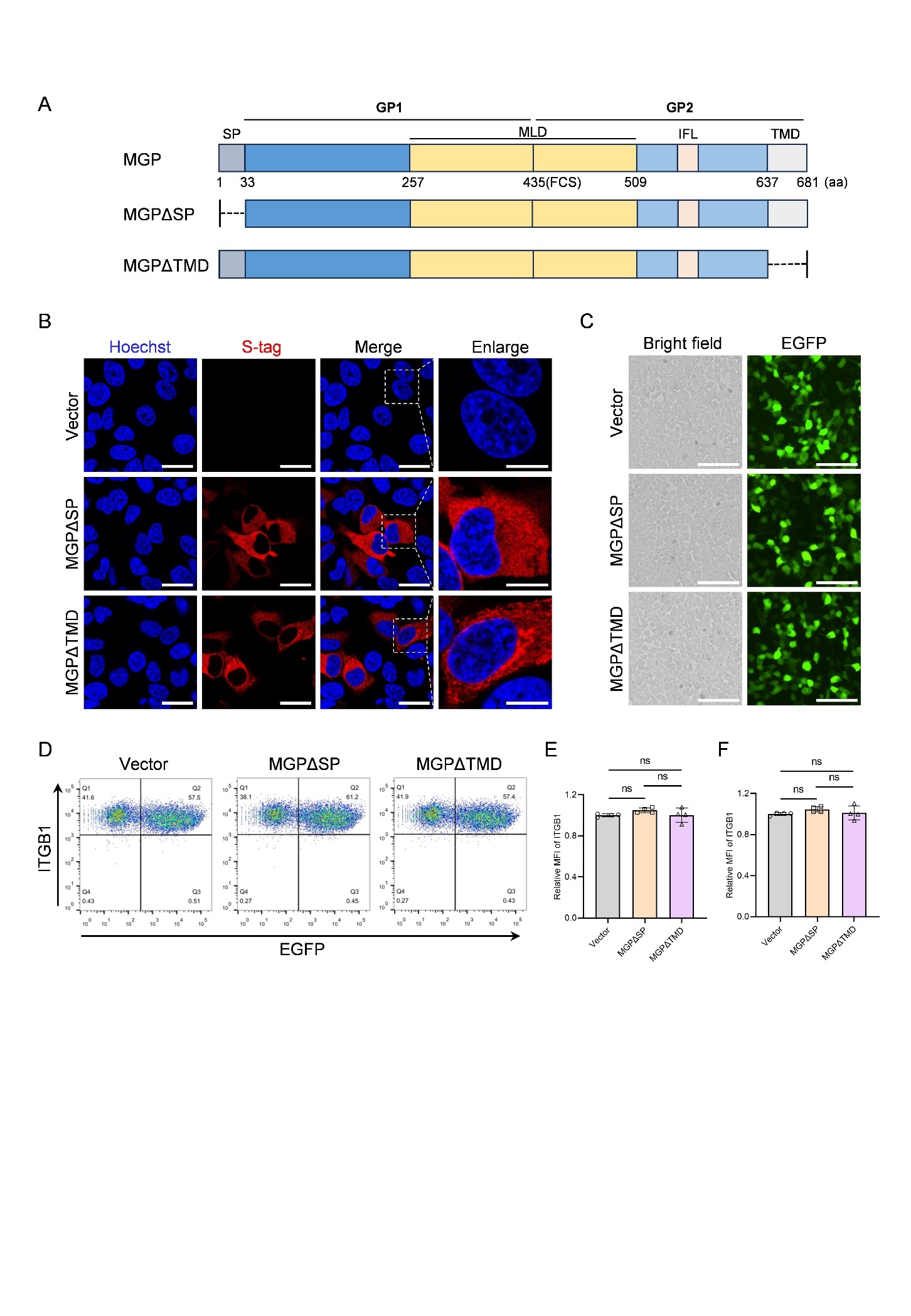
**

**Supplementary Figure S2. Cell membrane localization of MGP is essential for its cytopathogenic function.**

**A**. Schematic diagram illustrating the construction of MGP deletion mutants lacking the N-terminal signal peptide (MGPΔSP) and the transmembrane domain (MGPΔTMD). **B**. HeLa cells were transfected with a control vector or plasmids encoding the indicated proteins C-terminally tagged with S-tag. At 24 h post-transfection, cells were fixed and permeabilized, followed by IFA using an anti-S-tag antibody and confocal microscopy. Nuclei were stained with Hoechst (blue). **C-F**. HEK293T cells were co-transfection with the indicated protein expression plasmids or control vector along with pEGFP-N1, followed by visualization 24 h later under an inverse fluorescence microscopy (**C**) or by IFA with PE-conjugated anti-ITGB1 and FCM analysis (**D-F)**. FCM scatter plots (**D**) and relative MFI of cell surface protein signals in total cells (**E)** or EGFP positive cells (**F**) are respectively shown. Scale bars: 25 μm (10 μm in enlarged images) (**B**); 100 μm (**C**). Data are presented as means ± SD, n = 4 biological replicates. One-way analysis of variance (ANOVA) was used for multiple comparisons. ns, nonsignificant.

**
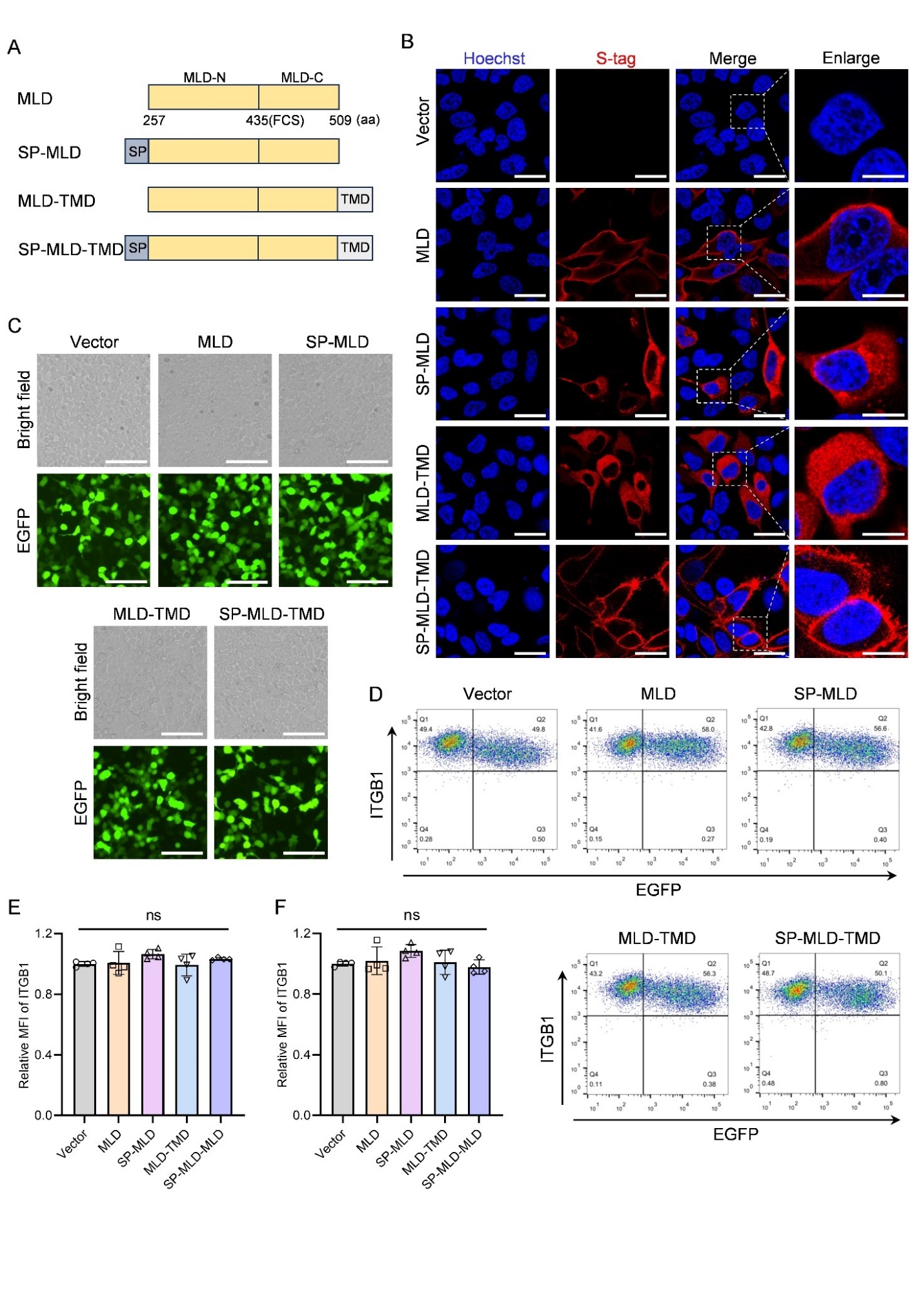
**

**Supplementary Figure S3.** **Expression of the MLD in isolation failed to induce any evident cytopathic effect.**

**A**. Schematic representation of various MLD expression constructs: the mucin-like domain (MLD) alone, MLD fused to the signal peptide (SP-MLD), the transmembrane domain (MLD-TMD), and both the SP and TMD (SP-MLD-TMD). **B**. HeLa cells were transfected with plasmids encoding the indicated proteins with a C-terminal S-tag or empty vector. At 24 h post-transfection, cells were fixed, permeabilized, and immunostained with anti-S-tag antibody, followed by confocal microscopy. Nuclei were counterstained with Hoechst. **C**. HEK293T cells were co-transfected with the indicated expression plasmids together with pEGFP-N1. Live-cell imaging was performed 24 h later using inverted fluorescence microscopy. **D-F**. HEK293T cells were co-transfected with the indicated protein expression plasmids or control vector along with pEGFP-N1, followed by IFA of nonpermeabilized cells with PE-conjugated anti-ITGB1 and FCM analysis. Scatter plots (**D**) and the relative MFI of cell surface protein signals in total cells (**E**) or EGFP positive cells (**F**) are respectively shown. Scale bars: 25 μm (10 μm in enlarged images) (**B**); 100 μm (**C**). Data are presented as means ± SD, n = 4 biological replicates. One-way analysis of variance (ANOVA) was used for multiple comparisons. ns, nonsignificant.

**
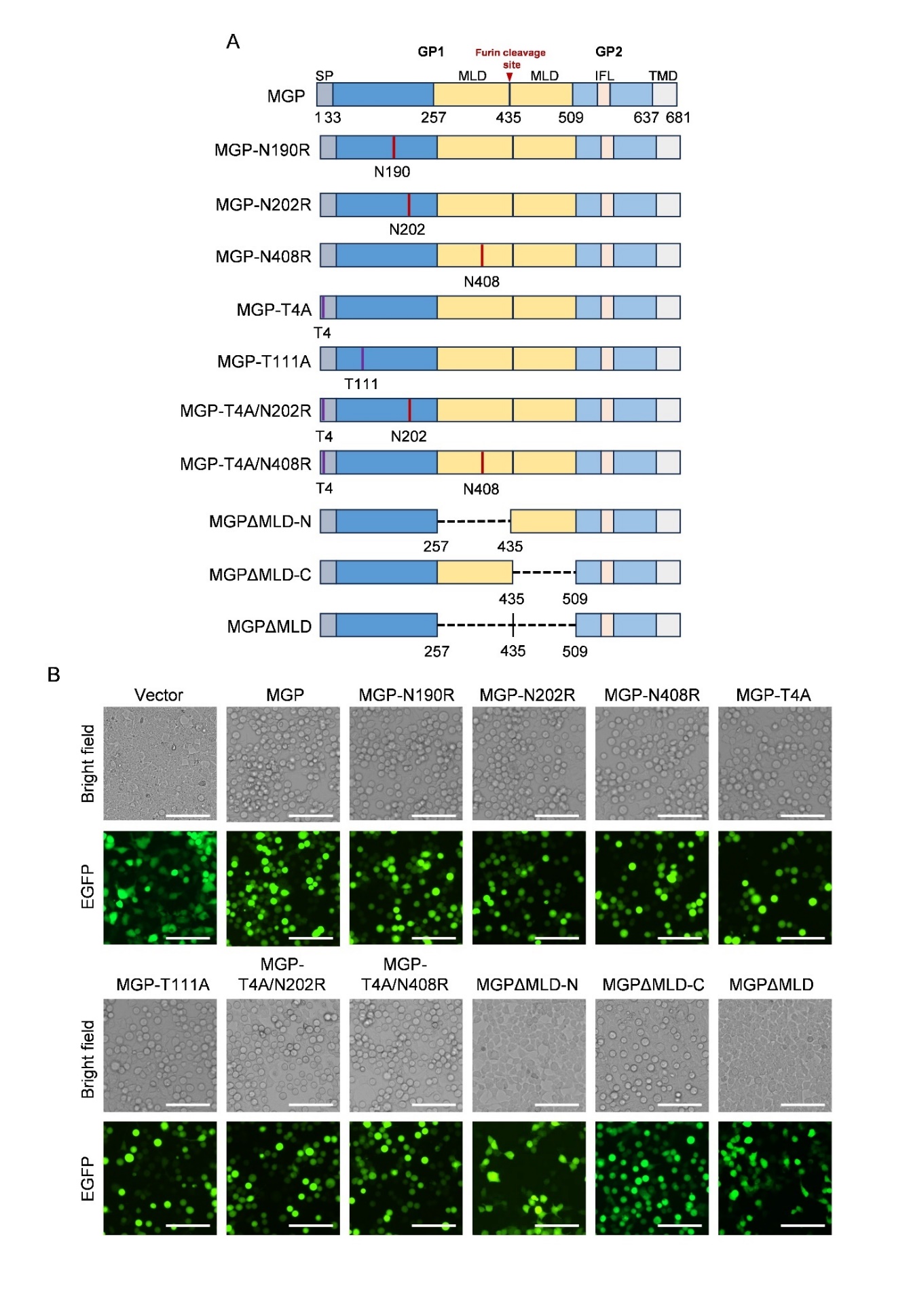
**

**Supplementary Figure S4. Effects of glycosylation site mutations and regional deletions on the cytopathogenic activity of MGP.**

**A**. Schematic diagram of MGP mutant and deletion construction. Several representative site-specific mutagenesis was used to inactivate N-glycosylation by changing Asparagine (N) to Glutamine (R), and O-glycosylation by changing Threonine (T) to Alanine (A). Some double-point mutants and fragment deletion mutants were also constructed. **B.** HEK293T cells were transfection with the indicated protein expression plasmids or control vector along with pEGFP-N1, followed by visualization 24 h later under an inverse fluorescence microscopy. Scale bars, 100 μm.

**
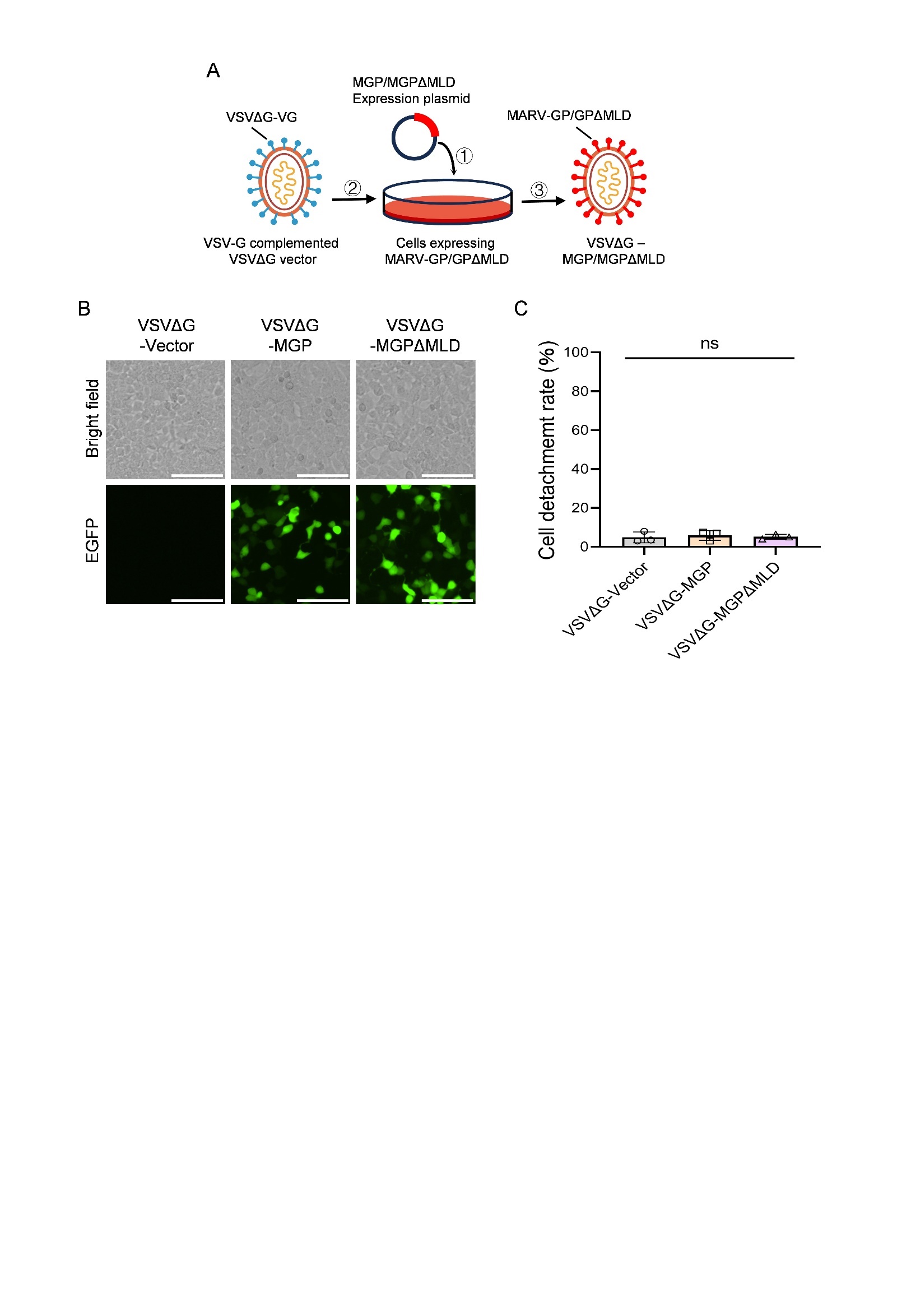
**

**Supplementary Figure S5.** **VSV pseudovirions displaying surface MGP or MGPΔMLD do not induce cell rounding or detachment.**

**A**. Schematic diagram of the generation of VSV pseudoviruses bearing MGP or MGPΔMLD. HEK293T cells were transfected with plasmids expressing MGP or MGPΔMLD. 12 h post-transfection, the cells were infected with VSVΔG-VG. Following a 2-h adsorption period at 37°C, the viral inoculum was removed, and the cells were further incubated for 24 h to harvest pseudoviruses bearing MGP or MGPΔMLD for subsequent experiments. **B**. HEK293T cells were transduced with the indicated pseudoviruses, followed by visualization 24 h later under an inverse fluorescence microscopy. Scale bars, 100 μm. **C**. Adherent and detached cells were collected 24 h after transfection, and the cell detachment rate was calculated. Data are presented as means ± SD, n = 3 biological replicates. One-way analysis of variance (ANOVA) was used for multiple comparisons. ns, nonsignificant.

**
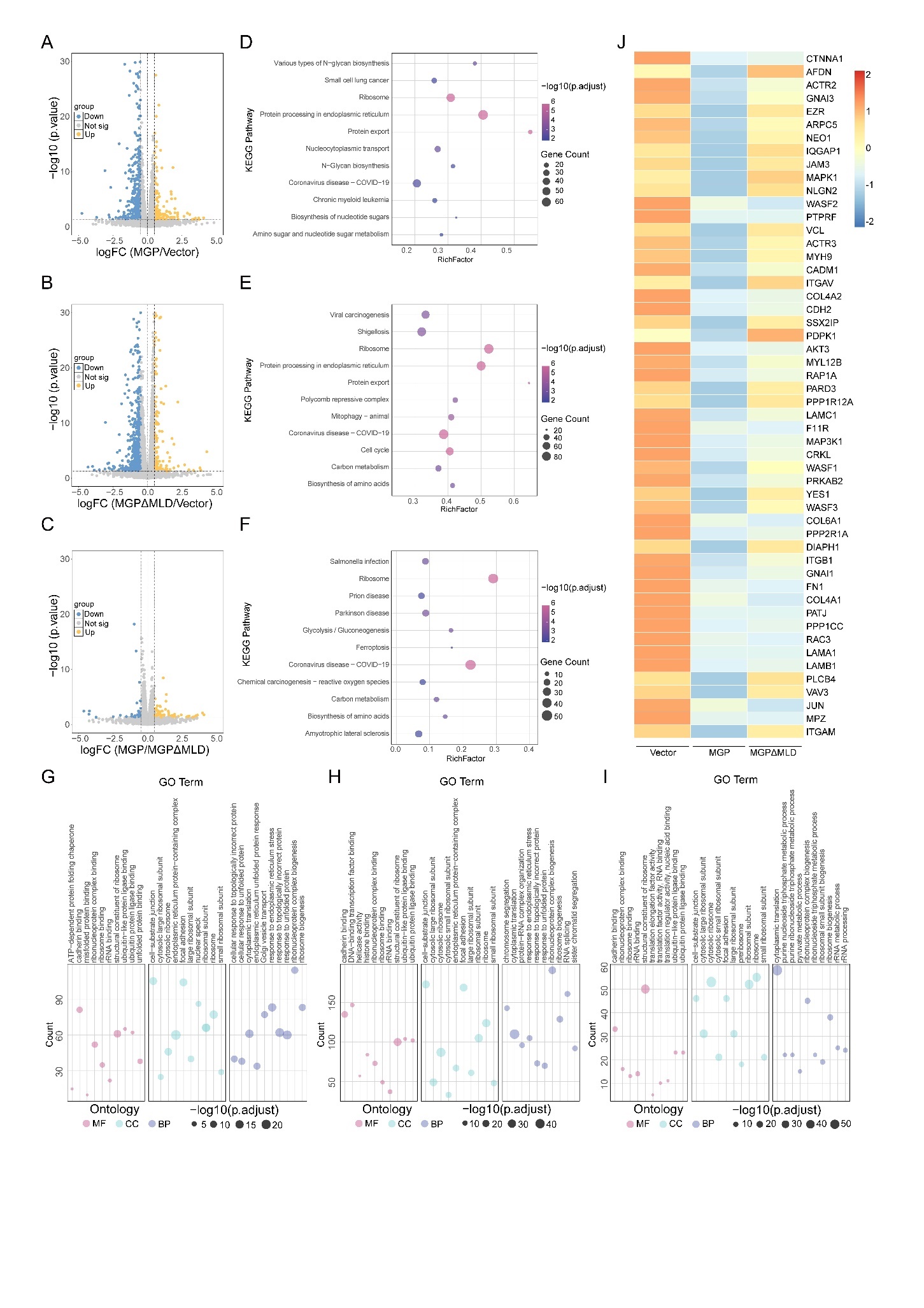
**

**Supplementary Figure S6. Effects of MGP and MGPΔMLD expression on cellular transcriptomes.**

HEK293T cells were transfected with the indicated expression plasmids or control plasmids. After 24 h, the cells were collected, and mRNA was extracted for transcriptome analyses. **A-C.** Volcano plot of differentially expressed genes between the groups. Each point represents an individual gene plotted with statistical significance (*p <* 0.05) and change in abundance. Upregulated (log_2_FC > 0.5) and downregulated (log_2_FC < -0.5) genes are colored yellow and blue, respectively. **D-F**. Bubble plot presenting functional enrichment of upregulated and downregulated DEGs detected between groups: MGP/Vector (**D**), MGPΔMLD/Vector (**E**), and MGP/MGPΔMLD (**F**). The size of the bubbles represents the number of DEGs, and the color represents the-log_10_(p.adjust) values. **G-I**. GO term (MF, molecular function; CC, cell component; BP, biological process) bubble plot representing functional enrichment of the genes from DEGs between groups: MGP/Vector (**G**); MGPΔMLD/Vector (**H**); MGP/ MGPΔMLD (**I**). The bubble size represents the-log_10_(p.adjust) values of the corresponding GO term. **J**. Identification of cell adhesion-associated genes downregulated by MGP in HEK293T cells. A heatmap displaying the gene expression levels (log_2_mean from three biological replicates) is shown for each condition.

**
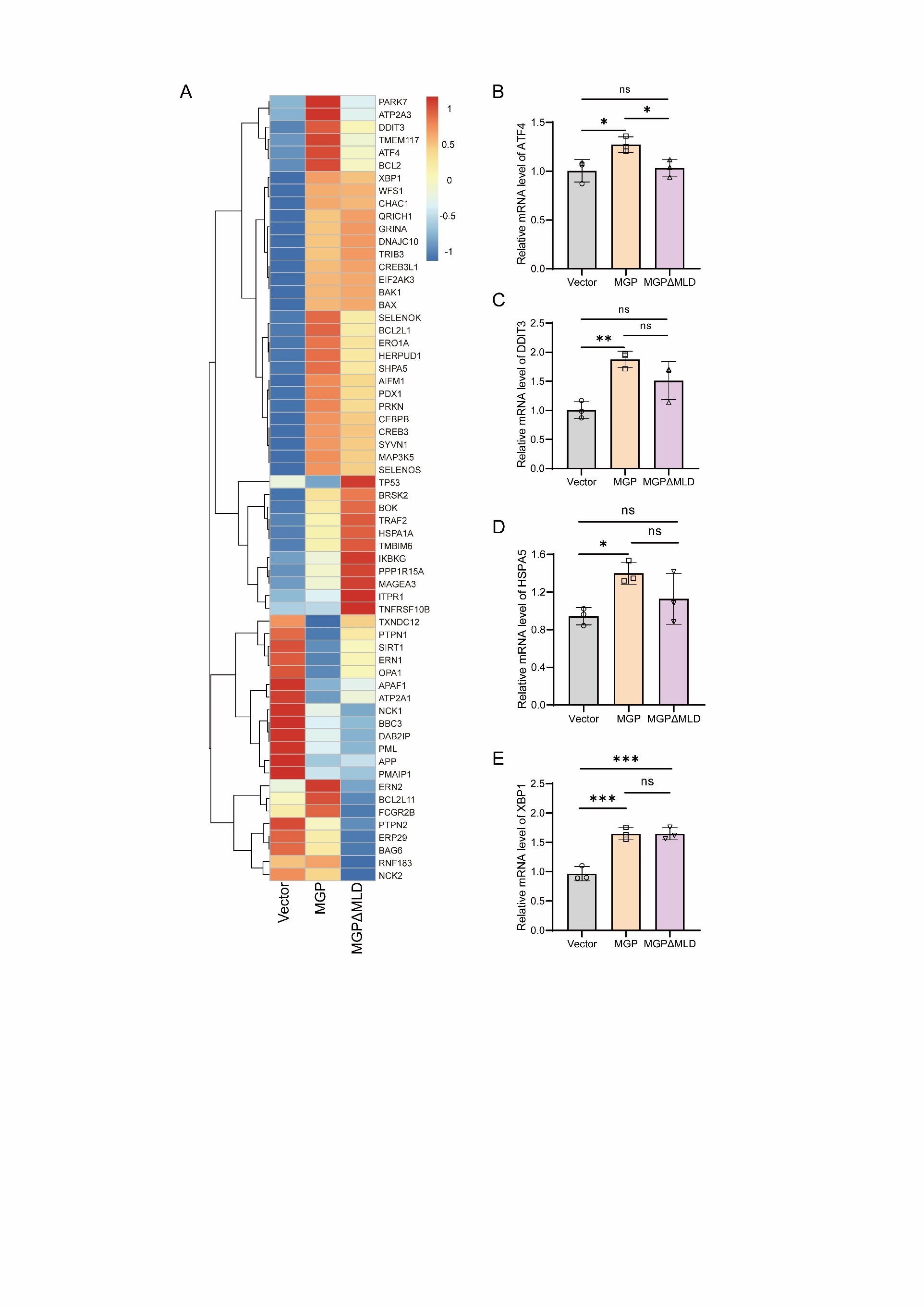
**

**Supplementary Figure** **S7. MGP regulates gene expression related to endoplasmic reticulum stress and the unfolded protein response.**

**A**. Heatmap displaying the log_2_FC for the key genes related to endoplasmic reticulum (ER) stress and unfolded protein response (UPR). Shown are the log2mean from three biological replicates. **B-E**, qPCR analyses of mRNA levels of representative genes. Data are presented as means ± SD, n = 3 biological replicates. One-way analysis of variance (ANOVA) was used for multiple comparisons. ****, *p* < 0.0001; ***, *p* < 0.001; **, *p* < 0. 01; *, *p* < 0. 05; ns, nonsignificant.

**
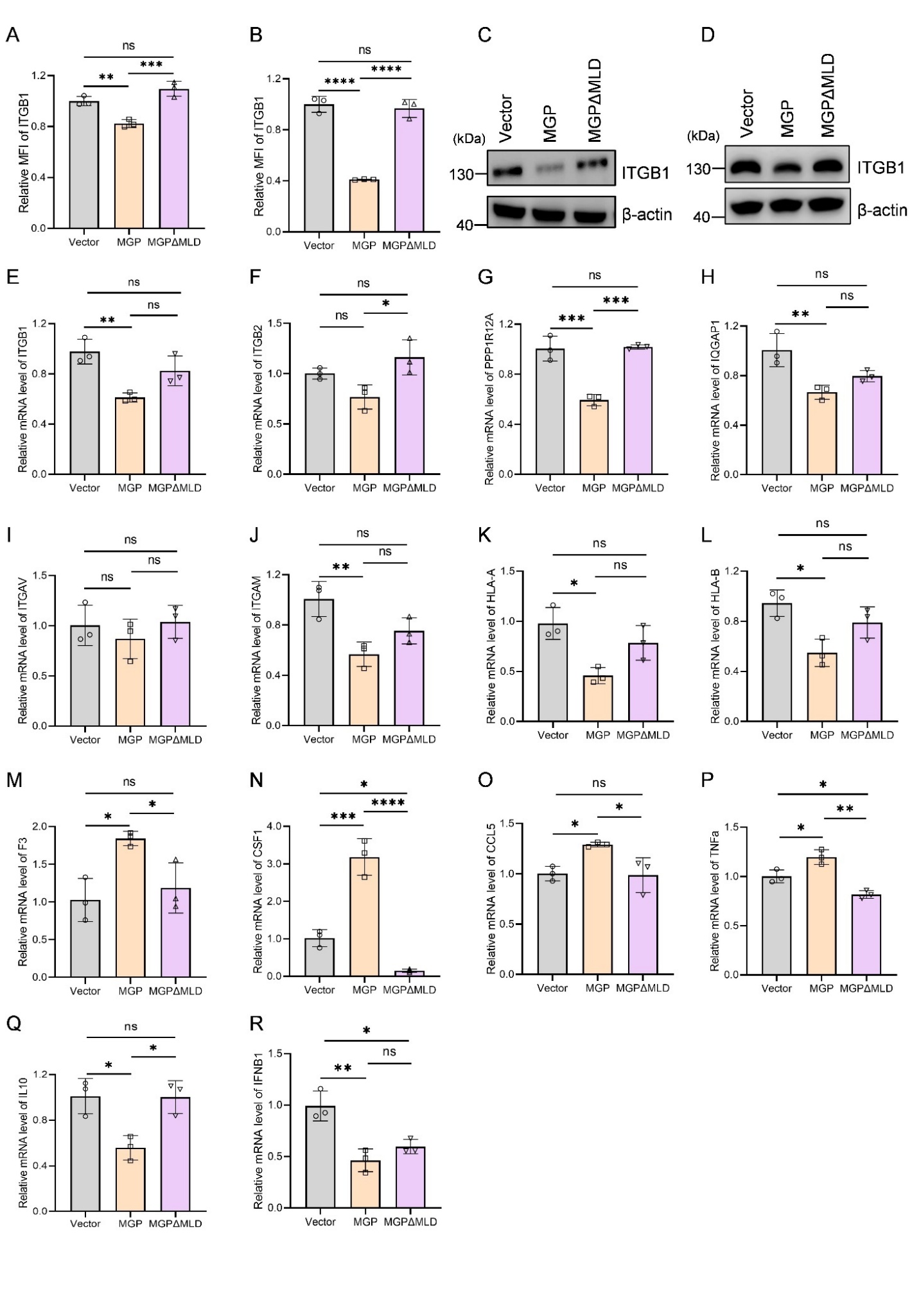
**

**Supplementary Figure S8.** **Differential regulation of cell surface proteins, endothelial adhesion molecules, immune factors, and pro-/anti-inflammatory cytokines by MGP and MGPΔMLD in endothelial and/or epithelial cells.**

**A-B**. MGP, but not MGPΔMLD, reduces detectable surface ITGB1 levels on HUVEC (endothelial) and Huh7 (epithelial) cells. HUVEC (**A**) and Huh7 (**B**) were transfected with the indicated plasmids, and ITGB1 signals were detected 24 h post-transfection by immunofluorescence staining of non-permeabilized cells followed by FCM. MFI results are shown. **C-D**. MGP, but not MGPΔMLD, downregulates total ITGB1 protein levels in HUVEC and Huh7 cells. HUVEC (**C**) and Huh7 (**D**) cells were transfected with the indicated plasmids, and total ITGB1 protein levels were analyzed 24 h post-transfection by Western blotting. **E-R**. Effects of MGP and MGPΔMLD on transcriptional expression of surface proteins, adhesion molecules, immune factors, and inflammatory cytokines in HUVEC cells. Cells were transfected as above, and the transcriptional expression levels of the indicated genes were analyzed 24 h post-transfection by qPCR. Data are presented as means ± SD, n = 3 biological replicates. One-way analysis of variance (ANOVA) was used for multiple comparisons. ****, *p* < 0.0001; ***, *p* < 0.001; **, *p* < 0. 01; *, *p* < 0. 05; ns, nonsignificant.

**
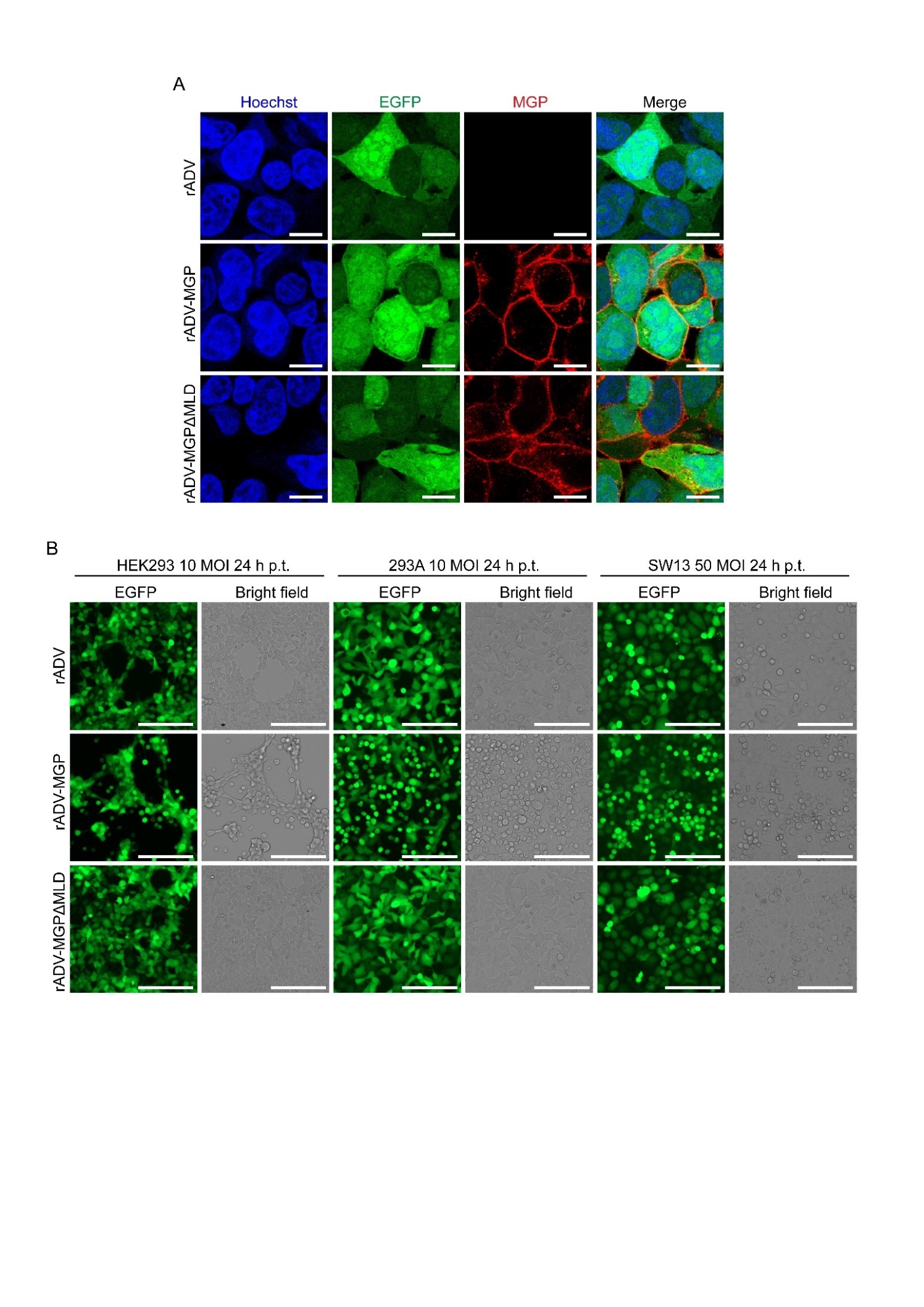
**

**Supplementary Figure S9. Expression of MGP, but not MGPΔMLD, by recombinant adenoviral** **transduction induces cell rounding/detachment.**

**A**. Expression and localization of MGP and MGPΔMLD. HEK239T cells were transduced with recombinant adenoviral vectors expressing EGFP as a reporter. At 24 h post-transduction, the cells were fixed and permeabilized, followed by IFA with an anti-MGP antibody and visualization by confocal microscopy. Nuclei stained with Hoechst are shown in blue. Scale bars, 25 μm. **B**. MGP, but not MGPΔMLD, induced cell rounding/detachment. HEK239 and HEK293A cells were transduced with recombinant adenovirus at 10 MOI and SW13 cells at 50 MOI. Cells were visualized 24 h later using an inverted fluorescence microscope. Scale bars, 100 μm.


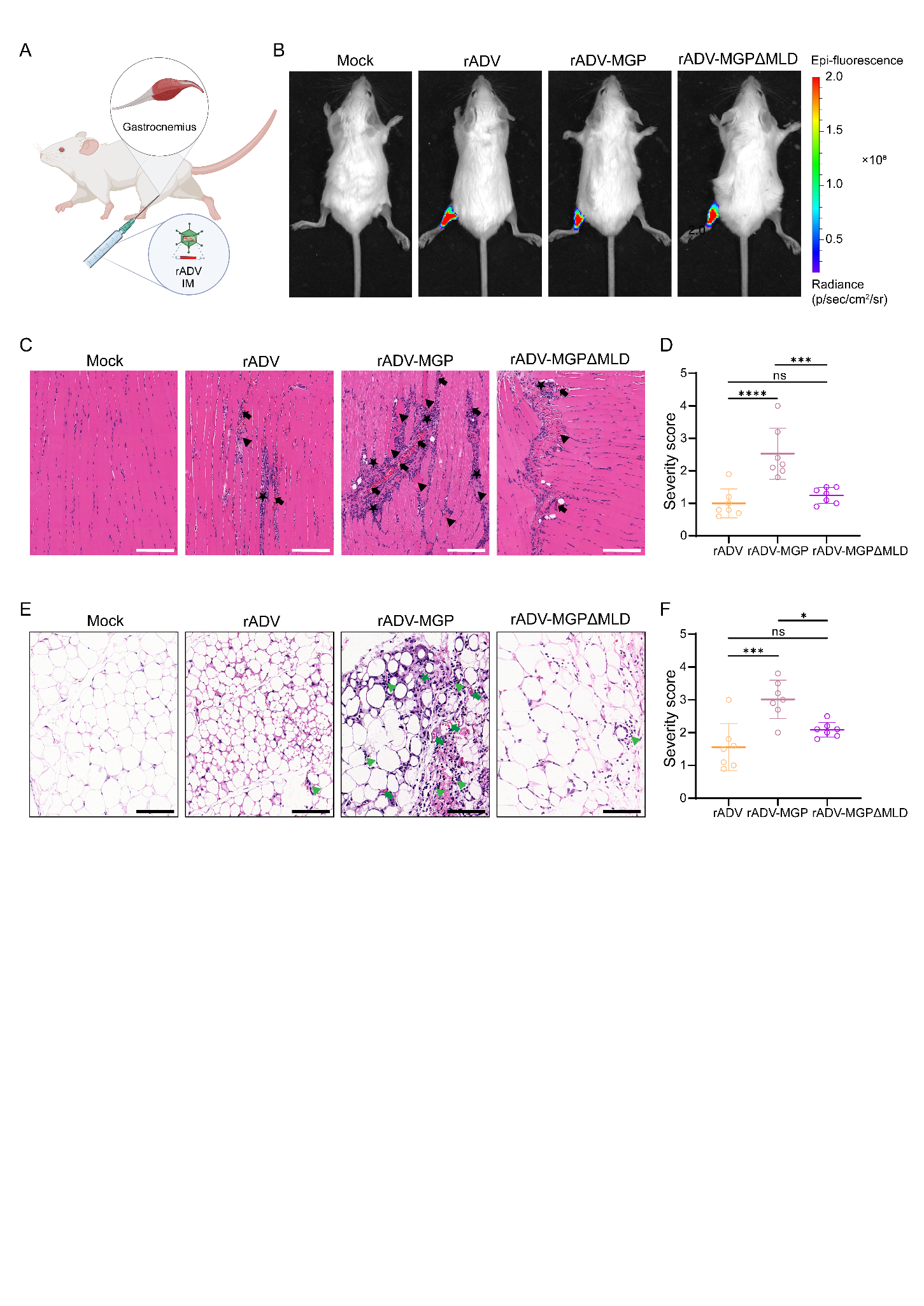


**Supplementary Figure S10. Mouse muscle transduction and injury model *in vivo*.**

**A**. Schematic representation of the mouse muscle transduction model. BALB/c mice were transduced with the indicated recombinant viral vectors (1×10^10^ TCID50/mL, 50 μL) via intragastrocnemius injection, followed by live imaging and histopathological analyses. Created in BioRender. Yao, T. (2026) https://BioRender.com/nmr2lpp. **B**. Live imaging of mice. After deep anesthesia, the EGFP fluorescence signal was monitored using an *in vivo* imaging system. Signal intensities at 72 h post-transduction is displayed using an RGB color scale. **C-F**. H&E staining of muscular (**C, D**) and connective tissues (**E, F**) after transduction. In the muscular tissues, arrows indicate bleeding or congestion in the muscle tissues; arrowheads indicate injury, disintegration, and necrosis of muscle fibers; and pentagrams indicate the aggregation of inflammatory cells (**C**). In the connective tissues, arrows indicate vessel congestion, arrowheads indicate inflammatory cell aggregation, and pentagrams indicate hyperplasia and fibrosis of the fascial tissue (**E**). Histopathological changes in the muscles and connective tissues were also assessed by scoring. Unduplicated images for each sample were selected, and the degree of muscle cell injury (**D**) or connective tissue inflammation (**F**) was scored as follows: 0, normal; 1, minimal change; 2, mild change; 3, moderate change; 4, marked change; and 5, severe change. Data are shown as mean ± SD, n = 7 mice per group (**D, F**). One-way analysis of variance (ANOVA) was used for multiple comparisons. ****, *p* <0.0001; ***, *p <* 0.001; *, *p <* 0.05; ns, nonsignificant. Scale bars: 200 μm (**C**); 100 μm (**E**).

**
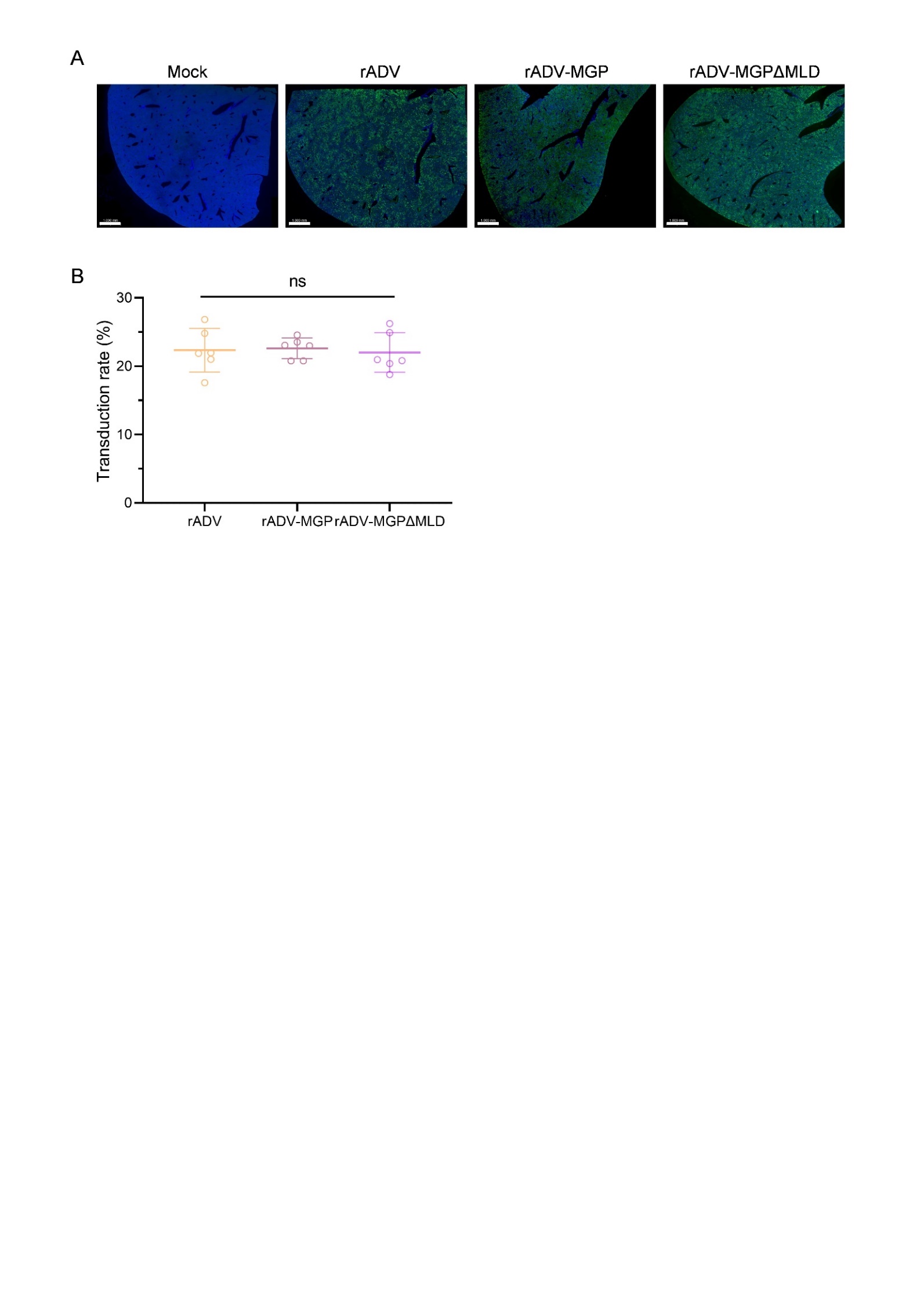
**

**Supplementary Figure S11. Assessment of recombinant adenovirus transduction efficiency in the murine liver.**

**A**. Panoramic view of the immunofluorescence of liver sections. Viral transductions were monitored with the anti-EGFP reporter antibody (green). Nuclei stained with Hoechst are shown in blue. Scale bars, 1 mm. **B**. The liver transduction efficiency was quantitatively analyzed. Data are presented as means ± SD, n = 6 mice/group. One-way analysis of variance (ANOVA) was used for multiple comparisons. ns, nonsignificant.

**
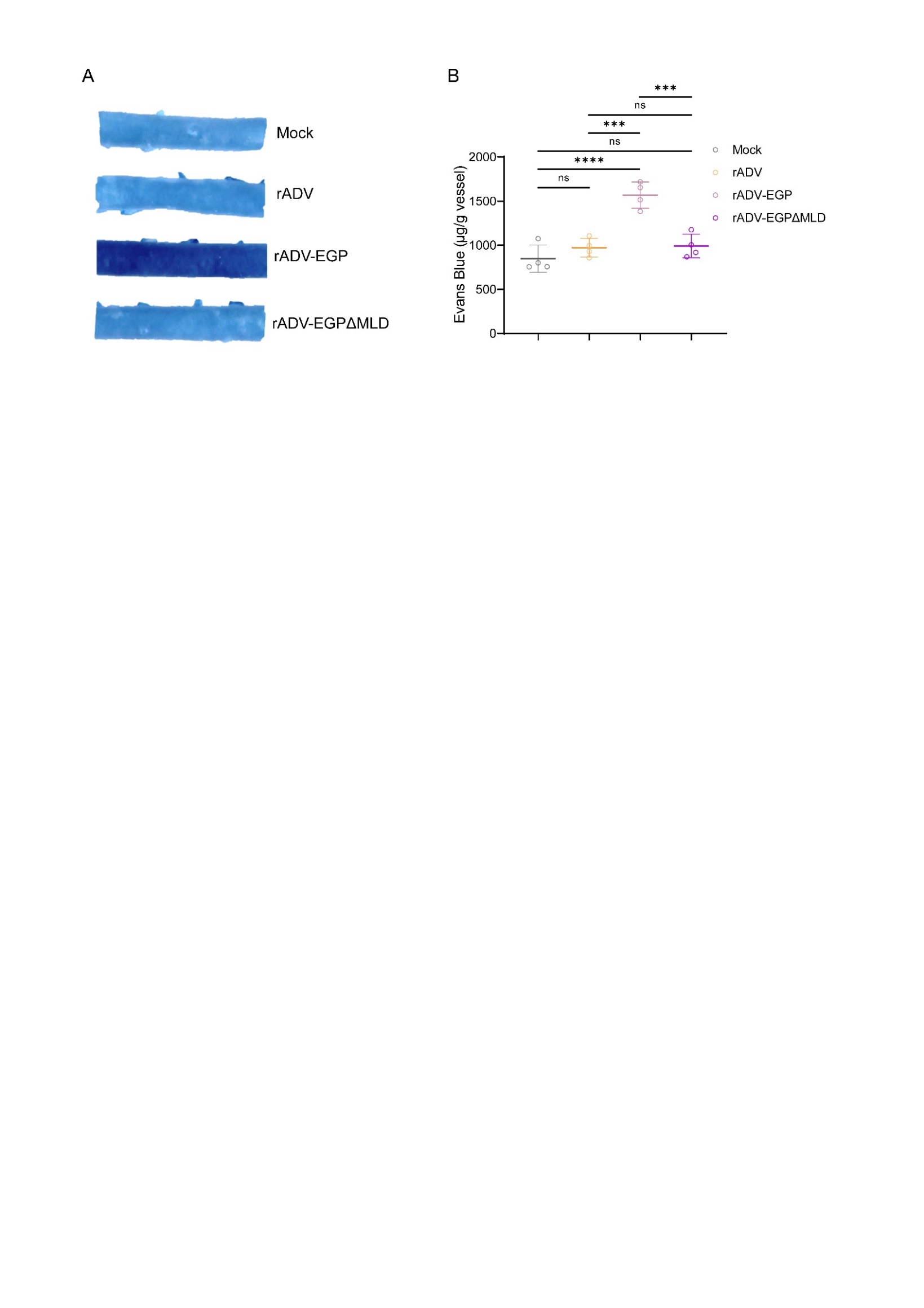
**

**Supplementary Figure S12. Validation of the vascular permeability-enhancing activity of EGP in the rat *ex vivo* vessel model established in this study.**

**A**. The rat aorta vessels cultured *ex vivo* were transduced with indicated adenoviral vectors. After 48 h, the permeability of the vessels was evaluated using Evans Blue staining. **B**. Evans Blue of the stained vessels was extracted with formamide and quantitatively analyzed using a standard curve method. EGP, but not the MLD-deleting mutant (EGPΔMLD) or control vector, significantly increases vascular permeability. Data are presented as means ± SD, n = 4 biological replicates. One-way analysis of variance (ANOVA) was used for multiple comparisons. ****, *p* < 0.0001; ***, *p* < 0.001; ns, nonsignificant.

**
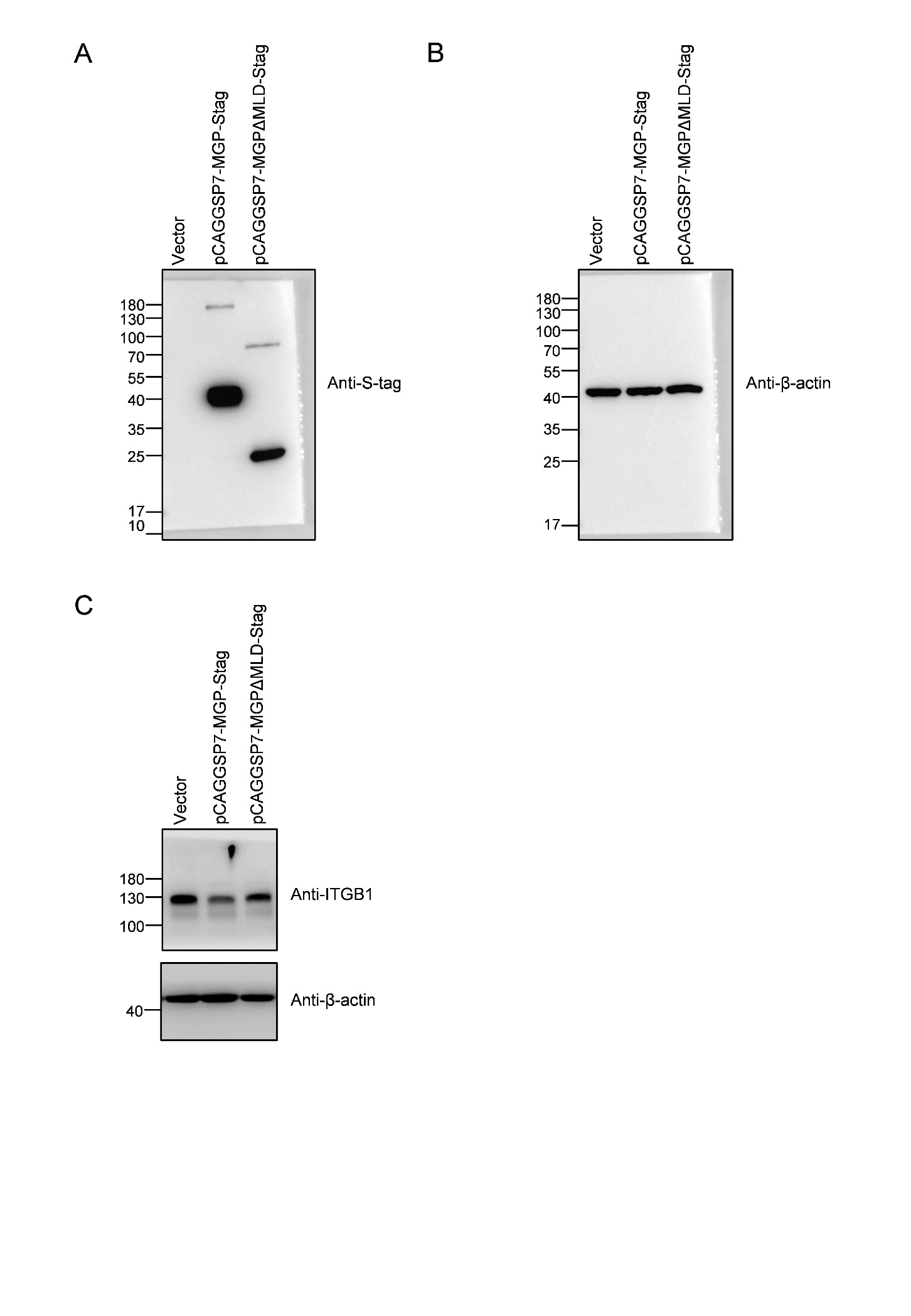
**

**Supplementary Figure S13. Original images** **for FIGURE 1A and 2I**

**A** and **B**. The original images of uncropped blots from FIGURE 1A. **C**. The original images for FIGURE 2I.

**
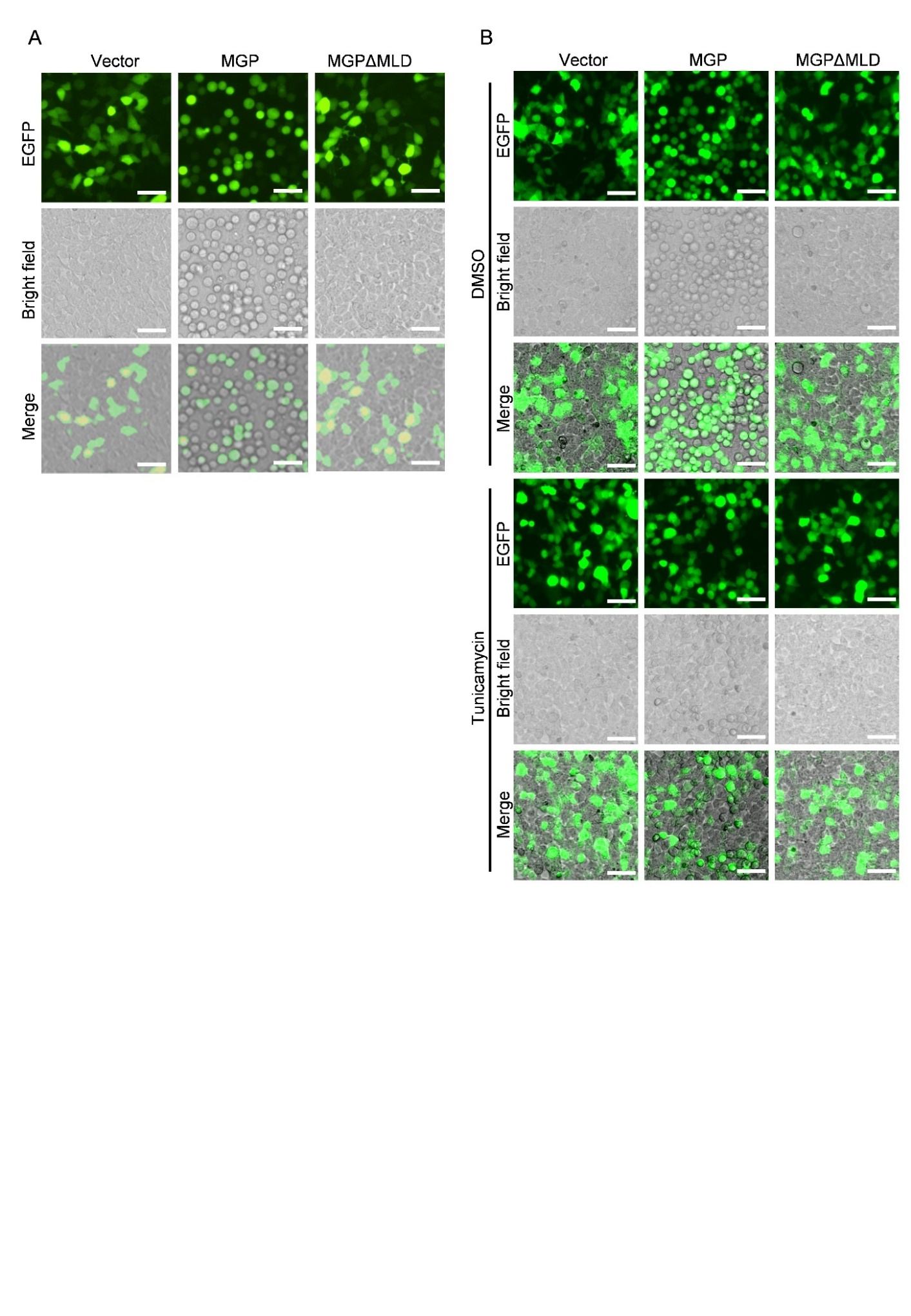
**

**Supplementary Figure S14.** **Merged images for FIGURE 1C and 2E**

**A**. Merged images from the main text FIGURE 1C. **B**. Merged images from the main text FIGURE 2E. Scale bars, 50 μm.

**Supplementary Table S1. Quantitative real-time PCR primers and probes.**

| **Name** | **Sequence (5’ to 3’)** |
| --- | --- |
| ITGB1-forward | CAAGAGAGCTGAAGACTATCCCA |
| ITGB1-reverse | TGAAGTCCGAAGTAATCCTCCT |
| IQGAP1-forward | AGCTGCAGTCTGGAGTGGAT |
| IQGAP1-reverse | CAGTGCTACTGCTGCCAATCT |
| ITGAV-forward | ATCTGTGAGGTCGAAACAGGA |
| ITGAV-reverse | TGGAGCATACTCAACAGTCTTTG |
| ITGAM-forward | GCTGCCGCCATCATCTTAC |
| ITGAM-reverse | GTGCCCTTGACATTAGCGTT |
| ATF4-forward | CTCCAACATCCAATCTGTCCCG |
| ATF4-reverse | TTCTCCAGCGACAAGGCTAAGG |
| DDIT3-forward | TTGCCTTTCTCCTTCGGGAC |
| DDIT3-reverse | AAGCAGGGTCAAGAGTGGTG |
| HSPA5-forward | CTGTCCAGGCTGGTGTGCTCT |
| HSPA5-reverse | CTTGGTAGGCACCACTGTGTTC |
| XBP1-forward | CCATGGGGAGATGTTCTGGAG |
| XBP1-reverse | CCTGGTTGCTGAAGAGGAGG |
| ITGB2-forward | TGCGTCCTCTCTCAGGAGTG |
| ITGB2-reverse | GGTCCATGATGTCGTCAGCC |
| PPP1R12A-forward | ACCGCATGACTACGCTCTTCTG |
| PPP1R12A -reverse | TTGAAGCGGACATTGGCAACCG |
| HLA-A-forward | GAGCGAGGCCGGTTCTCA |
| HLA-A-reverse | AACTGCTCCGCCTCATGG |
| HLA-B-forward | TAGCAGTTGTGGTCATCGGA |
| HLA-B-reverse | ACAGCTGTCTCAGGCTTTTCAA |
| F3-forward | ACTTGGCACGGGTCTTCTCCTAC |
| F3-reverse | TTGTTGGCTGTCCGAGGTTTGTC |
| CSF1-forward | TAGCATTGGGGGTGTTGTCTT |
| CSF1-reverse | AGCCACATGATTGGGAATGG |
| CCL5-forward | GGCAGCCCTCGCTGTCATCC |
| CCL5-reverse | GCAGCAGGGTGTGGTGTCCG |
| TNFα-forward | CCTCTCTCTAATCAGCCCTCTG |
| TNFα-reverse | GAGGACCTGGGAGTAGATGAG |
| IL10-forward | GACTTTAAGGGTTACCTGGGTTG |
| IL10-reverse | TCACATGCGCCTTGATGTCTG |
| IFNB1-forward | CACTACAGCTCTTTCCATGA |
| IFNB1-reverse | AGCCAGTGCTAGATGAATCT |
| EBOV-VP40-Probe | AAGCCTGGTTTCCAATTCGC |
| EBOV-VP40-forward | AGTTGGACTGGCGGAAGAAC |
| EBOV-VP40-reverse | CAGAGTCAATCGGCTGGGTC |
